# Multi-disturbance identification from mine wind-velocity data based on MSSW and WPT-GBDT

**DOI:** 10.1371/journal.pone.0284316

**Published:** 2023-04-18

**Authors:** Wentian Shang, Lijun Deng, Jian Liu, Yukai Zhou

**Affiliations:** 1 College of Safety Science and Engineering, Liaoning Technical University, Huludao, Liaoning, PR China; 2 Key Laboratory of Mine Thermal Power Disaster and Prevention of Ministry of Education, Liaoning Technical University, Huludao, Liaoning, PR China; TU Wien: Technische Universitat Wien, AUSTRIA

## Abstract

To overcome the false alarm problem that arises for mine wind-velocity sensors due to air-door and mine-car operation, a wind-velocity disturbance identification method based on the wavelet packet transform and gradient lifting decision tree is proposed. In this method, a multi-scale sliding window discretizes continuous wind-velocity monitoring data, the wavelet packet transform extracts the hidden features of discrete data, and a gradient lifting decision tree multi-disturbance classification model is established. Based on the overlap degree rule, the disturbance identification results are merged, modified, combined, and optimized. In accordance with a least absolute shrinkage and selection operator regression, the air-door operation information is further extracted. A similarity experiment is performed to verify the method performance. For the disturbance identification task, the recognition accuracy, accuracy, and recall of the proposed method are 94.58%, 95.70% and 92.99%, respectively, and for the task involving further extraction of disturbance information related to air-door operation, those values are 72.36%, 73.08%, and 71.02%, respectively. This algorithm gives a new recognition method for abnormal time series data.

## Introduction

During the normal production period of a mine, air-door and mine-car operations are necessary to satisfy the production demand; however, these operations cause abnormal fluctuations in the wind-velocity sensor data. The air door is an important structure in the mine, and its main function is to isolate the wind flow in the tunnel and to guide the wind-flow direction [1, 2]. Mine cars constitute important mine transportation equipment and are characterized by flexibility and a wide application range. They are responsible for transportation of ore, waste rock, equipment, and people within the mine [3, 4]. However, the wind-flow structure changes caused by air-door operation, as well as the weak piston effect caused by mine-car operation, generate abnormal fluctuations with time delays and chaotic fluctuation ranges in the wind-velocity monitoring data for the relevant tunnel or for associated tunnels affected by the turbulent pulsation of the wind flow itself [5, 6]. Because these abnormal fluctuations are similar to power quality signal disturbances, the air-door and mine-car production activities are collectively referred to as “wind-velocity disturbances.”

When a disturbance generates abnormal fluctuations in wind-velocity data, the monitoring system output can easily be misinterpreted as indicating incidents such as tunnel collapses and air-door damage; thus, false alarms can arise, causing resource wastage or property loss. Therefore, disturbance identification and further information extraction for abnormal wind-velocity data fluctuations caused by various disturbances can improve production efficiency and reduce loss. A wind-velocity sensor generates time-series data, and disturbance-induced wind-velocity data fluctuations constitute abnormal time-series data; thus, any technique for identifying a disturbance from the associated wind-velocity sensor monitoring data essentially corresponds to an abnormal time-series detection method.

Existing time-series anomaly monitoring methods mainly utilize data preprocessing, feature extraction, and recognition methods. In terms of data preprocessing, data standardization and discretization are two common techniques, and the common data standardization methods include deviation standardization [7], standard deviation standardization [8], entropy difference standardization [9], and range standardization proportion [10]. Among these methods, deviation standardization is most suitable for wind-velocity monitoring data that is mostly stationary, with only a small range of abnormal fluctuations. Common data discretization methods include equidistance discretization [11], equifrequency discretization [12], sliding window discretization [13], and k-means clustering algorithm discretization [14]. Sliding window discretization is often used in object recognition tasks and has yielded better recognition results than other methods. Some improved methods based on continuous application of sliding window discretization have been proposed; these methods use adaptive sliding windows [15], multi-scale sliding windows (MSSWs) [16], and semi-fixed length sliding windows [17], for example. Among these techniques, the data discretized by the MSSW have a large redundancy; thus, this technique is only suitable for time-series datasets containing small volumes of data.

Commonly used feature extraction methods are often based on the Fourier transform [18], wavelet transform (WT) [19], sparse representation [20], empirical mode decomposition [21], or other signal processing technologies. The WT is suitable for analyzing the transient characteristics of a sudden signal and has been widely used for power quality transient-disturbance identification and detection, power quality disturbance data compression, and signal reconstruction [18, 19]. On the basis of the discrete wavelet transform (DWT) and other WTs, wavelet packet (WPT) technology has been developed to overcome the poor high-frequency signal resolution yielded by WTs [22].

As regards recognition methods, artificial intelligence (AI) is being incorporated in an increasing number of studies aiming to improve anomaly recognition algorithm performance. Commonly used AI and machine learning models include the naive Bayes algorithm [23], support vector machine (SVM) [24], gradient lifting decision tree (GBDT) [25], and back propagation neural network (BP) [26]. In particular, the GBDT model is a decision tree algorithm based on a gradient lifting framework, which inherits the advantages of the statistical model and AI method. Compared with other machine learning models, the GBDT model has the advantages of high prediction accuracy and good processing of low-dimensional data.

In this study, the advantages of the deviation standardization, MSSW, WPT, and GBDT techniques are combined with the basic disturbance-induced wind-velocity variation law to construct a multi-disturbance identification method referred to as “MSSW-based WPT-GBDT.”

First, the wind-velocity time-series data collected by a single sensor are preprocessed using deviation standardization and an MSSW and then transformed into sub-time-series data within a unified variation range, which is convenient for subsequent feature extraction. Then, a statistical method is employed to extract some features, and WPT is used to extract the multilayer high- and low-frequency coefficients of each sub-time-series data. By calculating the entropy of the high- and low-frequency coefficients of each layer, more of the features are obtained. Then, GBDT is used to learn and identify the wind-velocity monitoring data characteristics under different disturbance types and conditions. Finally, the recognition results are combined and selected based on the intersect over union (IoU) (i.e., the overlap degree) and confidence degree, and are used to achieve high average recognition accuracy and generalization performance. In addition, this algorithm gives a new recognition method for abnormal time series data.

A similarity experiment and change rule for air-door operation were introduced and discussed in detail in the Lijun Deng’s previous studies [1, 2]. Therefore, in the present study, only the mine-car operation similarity experiment, the MSSW-based WPT-GBDT mine multi-disturbance recognition algorithm, and the verification tests are presented. The verification tests confirm the efficacy of the proposed multi-disturbance recognition algorithm when applied to mine wind-velocity data.

## Mine-car operation similarity experiment

### Experimental model design

**Fig 1:**
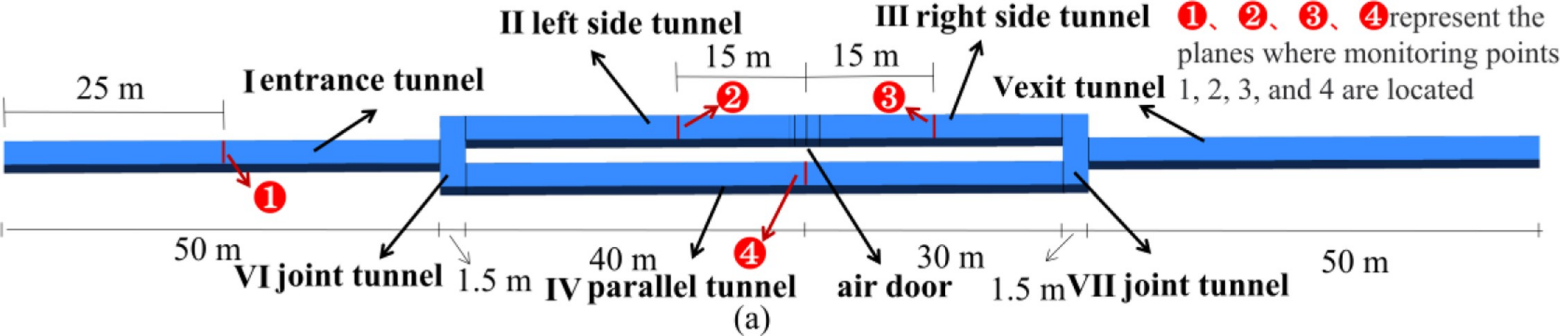
Overall tunnel dimensions and monitoring-point locations.

**Fig 2:**
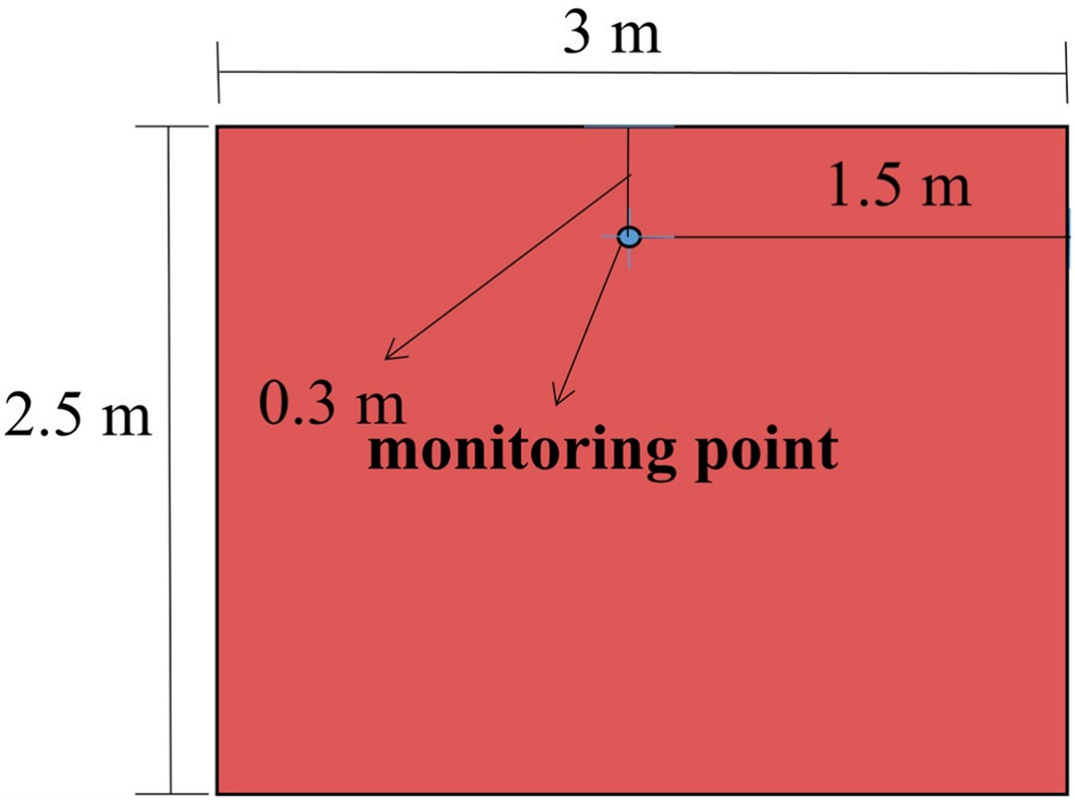
Cross section showing monitoring-point location.

**Fig 3:**
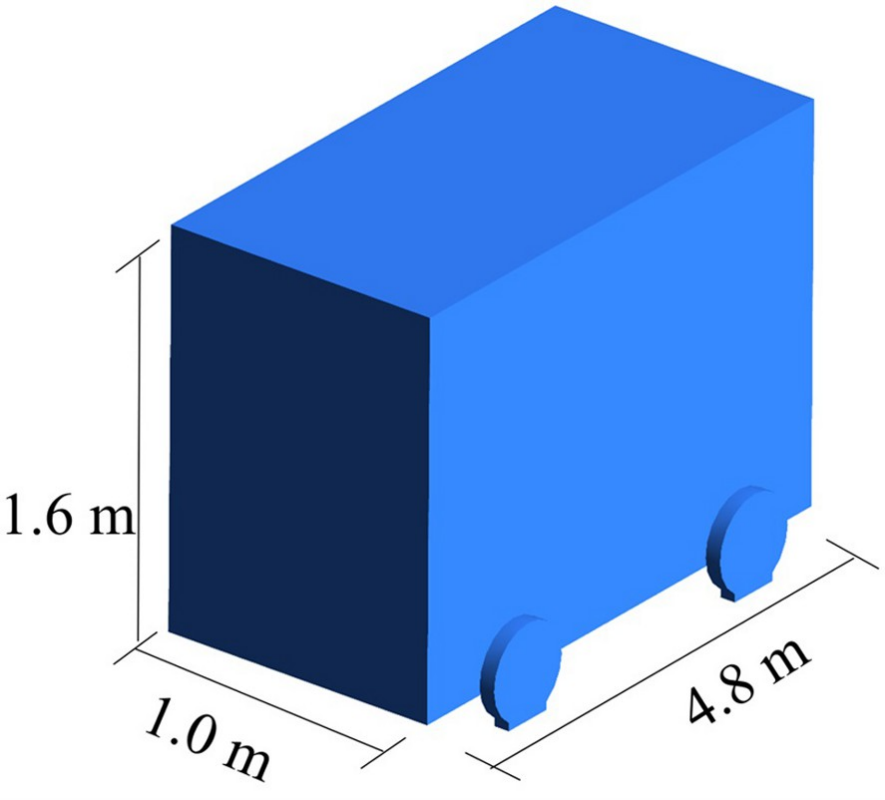
Mine-car dimensions.

The experimental model employed in this study is based on the experimental tunnel at the Key Laboratory of Mine Thermal Power Disaster and Prevention of the Ministry of Education of Liaoning University of Engineering and Technology. The detailed dimensions of the tunnel, the cross sections in which the tunnel monitoring points are located, and the mine car are shown in Figs 1–3, respectively.

**Fig 4:**
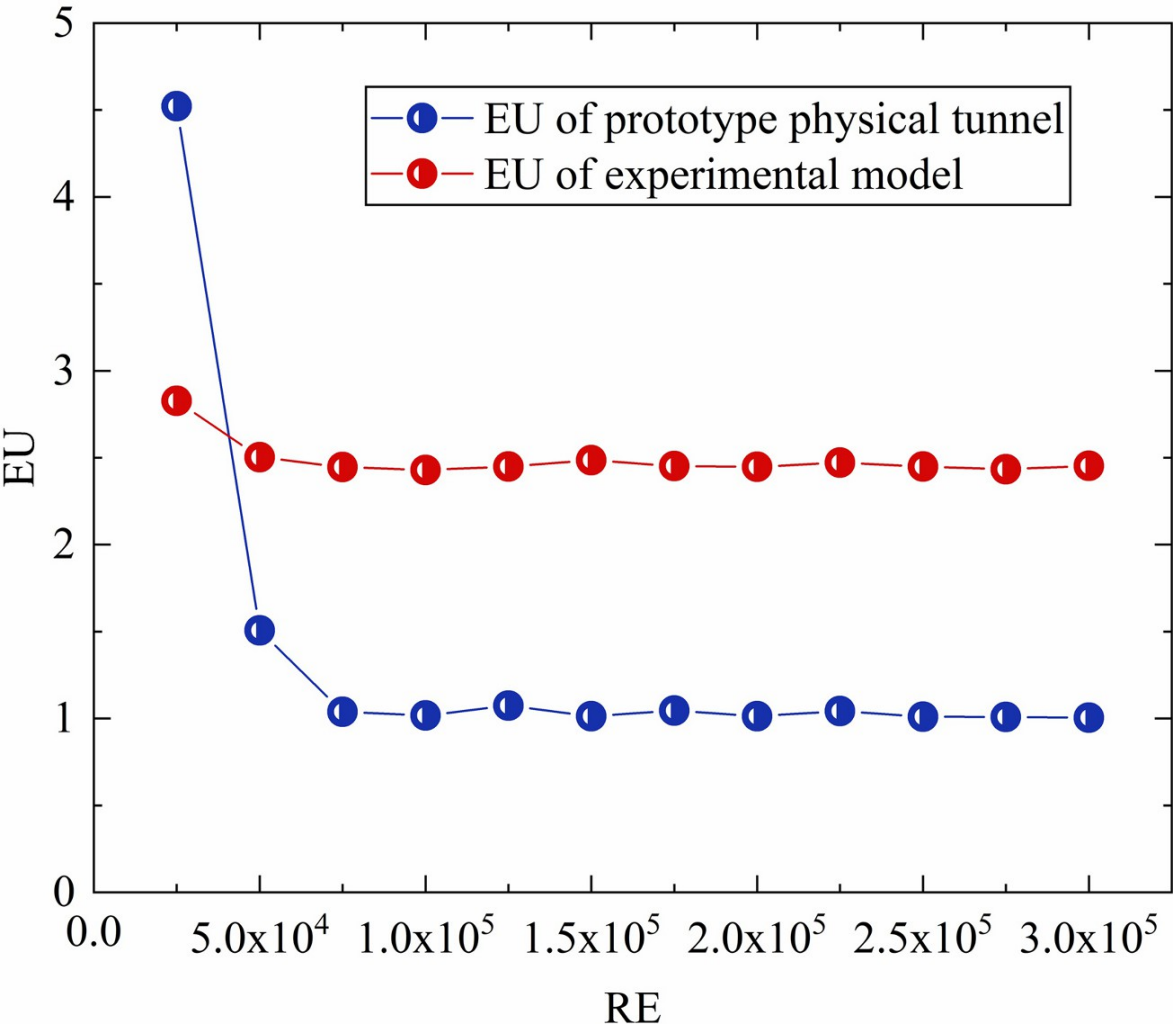
Comparison of EU vs. RE trends for real and experimental models.

When designing the experimental model, it was necessary to achieve flow similarity between the experimental model and the prototype physical tunnel shown in Fig 1; that is, to achieve geometric, motion, and dynamic similarity, where motion similarity manifests from the geometric and dynamic similarities [27, 28]. The prototype tunnel is incorporated in a relatively long and thin structure; therefore, to conduct a similar model experiment with geometric similarity, we must adopt different scales in different directions. Thus, a variability model is required [28]. In this study, the length variation of the designed experimental model was 2 and the overall similarity scale was 1:16. As regards dynamic similarity, in two geometrically similar models where the Euler number (EU) is independent of the Reynolds number (RE), the flow field enters the second self-simulation region; this can satisfy the motion similarity requirement [27, 28]. Numerical simulations were performed to simulate different RE flow fields in the two models, i.e., the experimental model and the prototype physical tunnel, and EU values under different REs were calculated, as shown in Fig 4.

**Fig 5:**
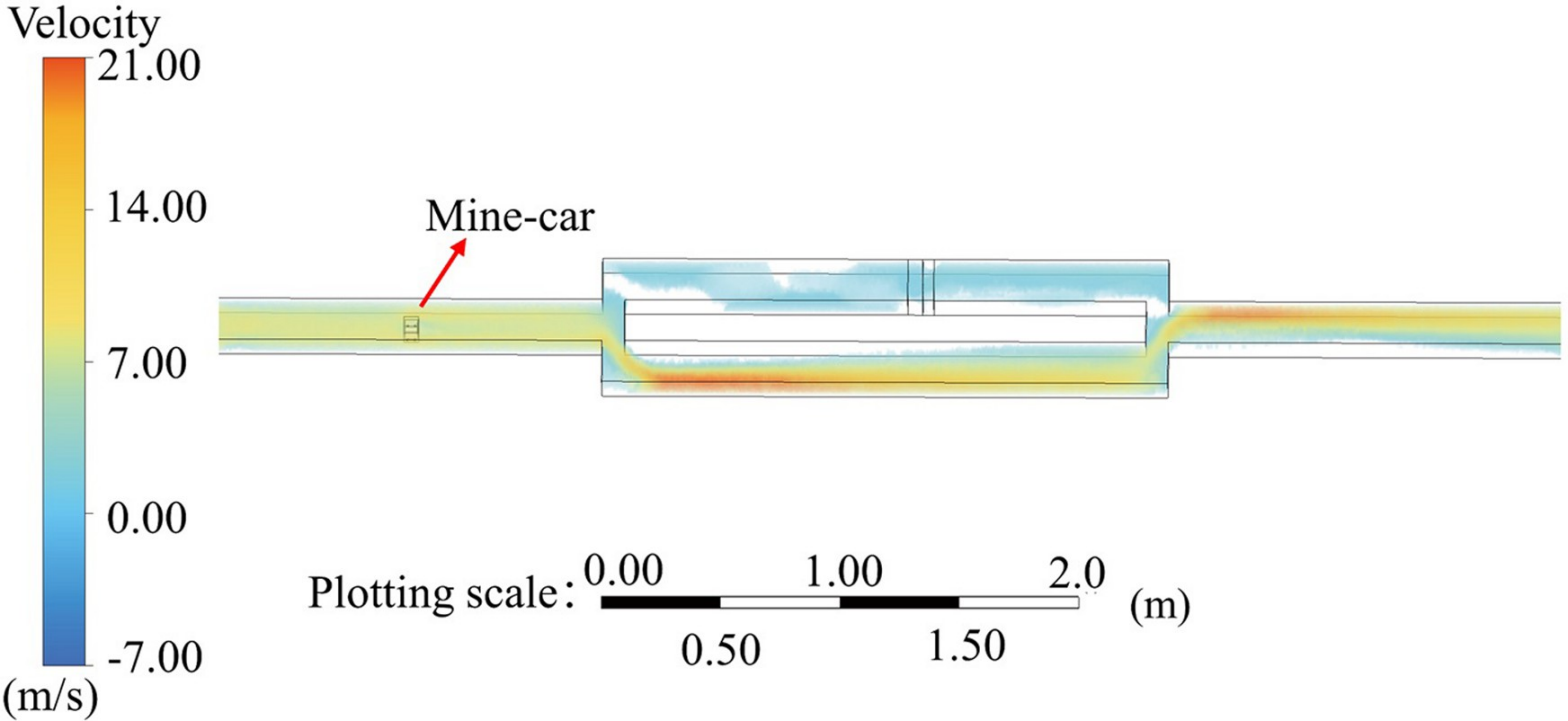
Experimental-model numerical simulation parameters and flow-field distribution for 7.90-m/s inlet wind velocity.

**Fig 6:**
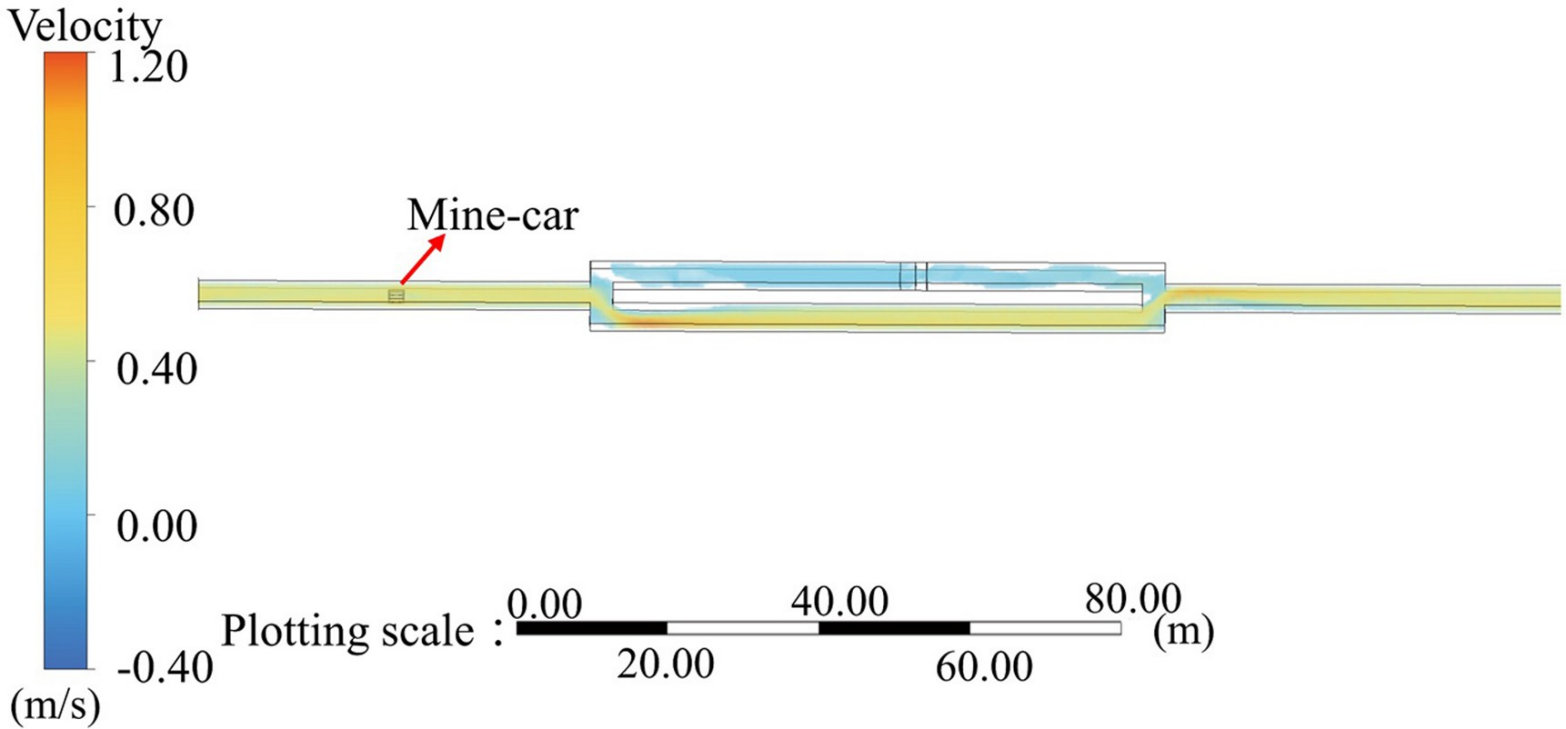
Physical-model numerical simulation parameters and flow-field distribution for 0.49-m/s inlet wind velocity.

As apparent from Fig 4, when RE exceeded 0.75 × 10^5^, the flow-field EU values for the original and experimental models did not change greatly. That is, when the wind velocity in the experimental model exceeded 7.9 m/s, the flow field was in the same second self-contact zone as the physical-model flow field when the wind velocity exceeded 0.49 m/s; this characteristic ensured similar flow-field movement for the two models. A numerical simulation with specific parameter settings for the two models was performed, and the flow-field distribution when the experimental-model inlet wind velocity was 7.9 m/s was compared with that for the physical model when the inlet wind velocity was 4.9 m/s, as shown in Figs 5 and 6, respectively. The specific distributions of the two flow fields were similar.

### Experimental device and working conditions

**Fig 7:**
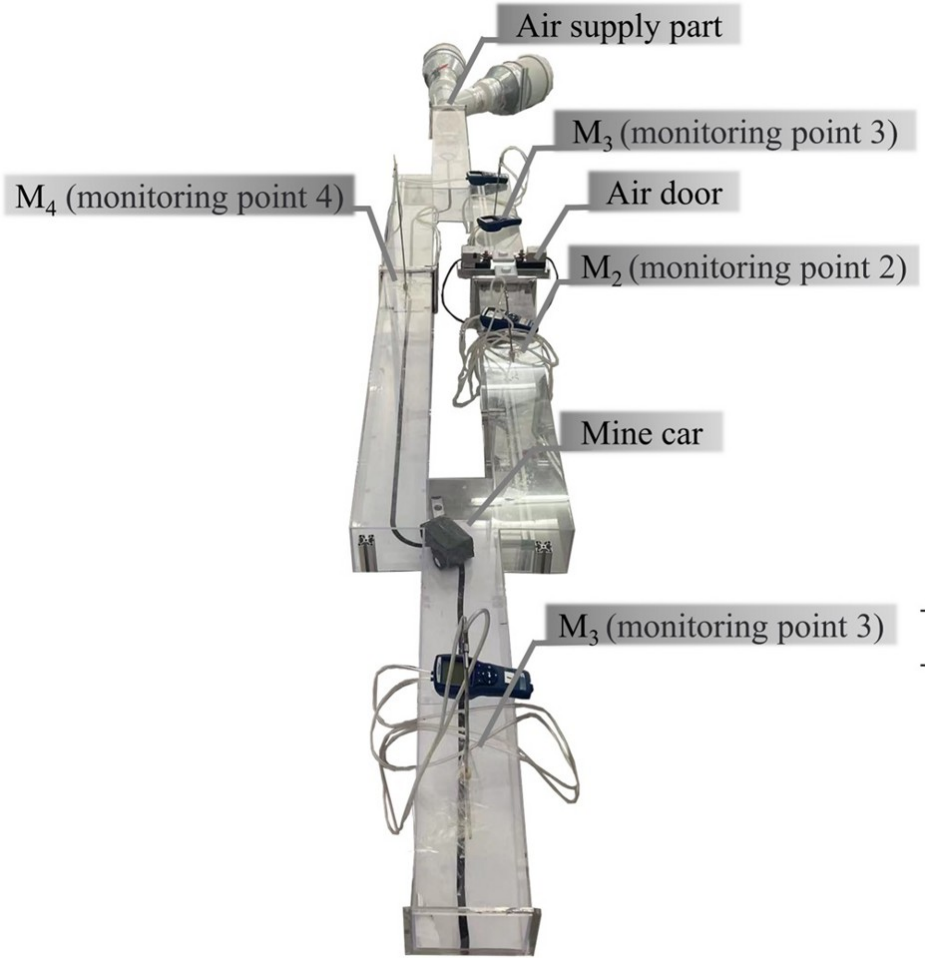
Overview of experimental model.

**Fig 8:**
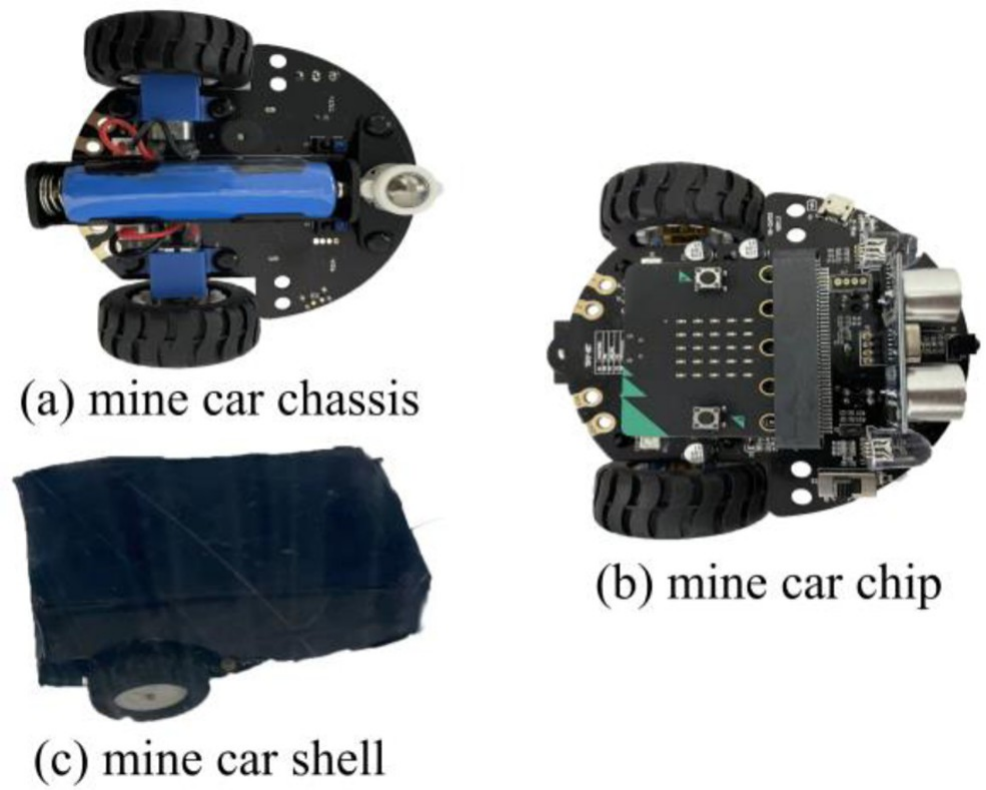
Detailed views of mine car.

Based on the analyses described in the previous subsection, the air supply required for the specific model size was determined, along with other parameters pertaining to the experimental devices required for the similarity experiment. Hence, we designed the experimental model and mine-car device shown in Figs 7 and 8, respectively. The mine car had a Raspberry PI computer and, using a program in the Python language, Bluetooth control, infrared line tracking, automatic obstacle avoidance, velocity adjustment, and other functions were achieved.

Four constituent factors impacted the specific working conditions of the mine car: the running route (1), velocity (2), direction (3), and inlet wind velocity (4). These four factors varied with the mine-car model and the tasks performed. Therefore, experiments were performed for 100 working conditions, which were combinations of the specific parameters of the four component factors listed in Table 1.

**Table 1:** Mine-car operation experimental conditions.

Factor	No.	Unit	Specific parameters
**Route**	1	‒	A→B→A, C→D→C
**Mine car running velocity**	2	m/s	0.06, 0.07, 0.08, 0.09, 0.10
**Direction**	3	‒	With wind, Against wind
**Inlet wind velocity**	4	m/s	8.00, 8.50, 9.00, 9.50, 10.00

**Fig 9:**
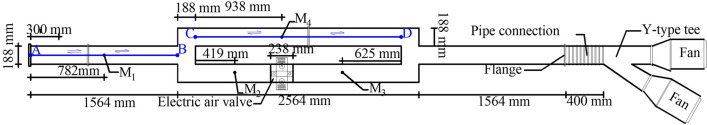
Mine-car operation routes.

The two routes (A→B→A, C→D→C) in Table 1, which were considered for Factor 1, are shown in Fig 9. The three velocities considered for Factor 2 were obtained through scaling according to a geometric similarity of 1:16 and for a length direction with variability 2. Before scaling, the three mine-car running velocities were 2, 2.5, and 3 m/s, respectively.

### Comparison of experimental data under different working conditions

**Fig 10:**
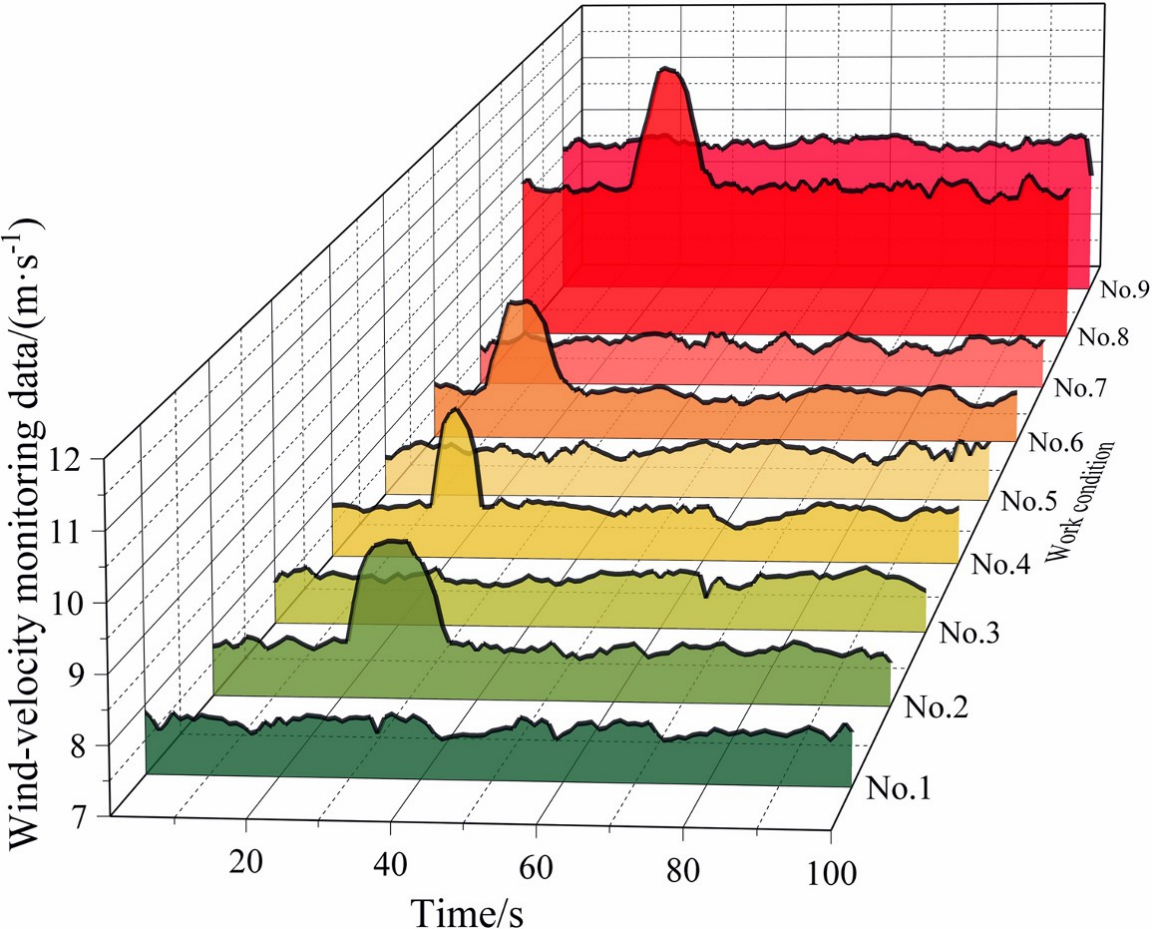
Wind-velocity data recorded at monitoring Point 1.

**Fig 11:**
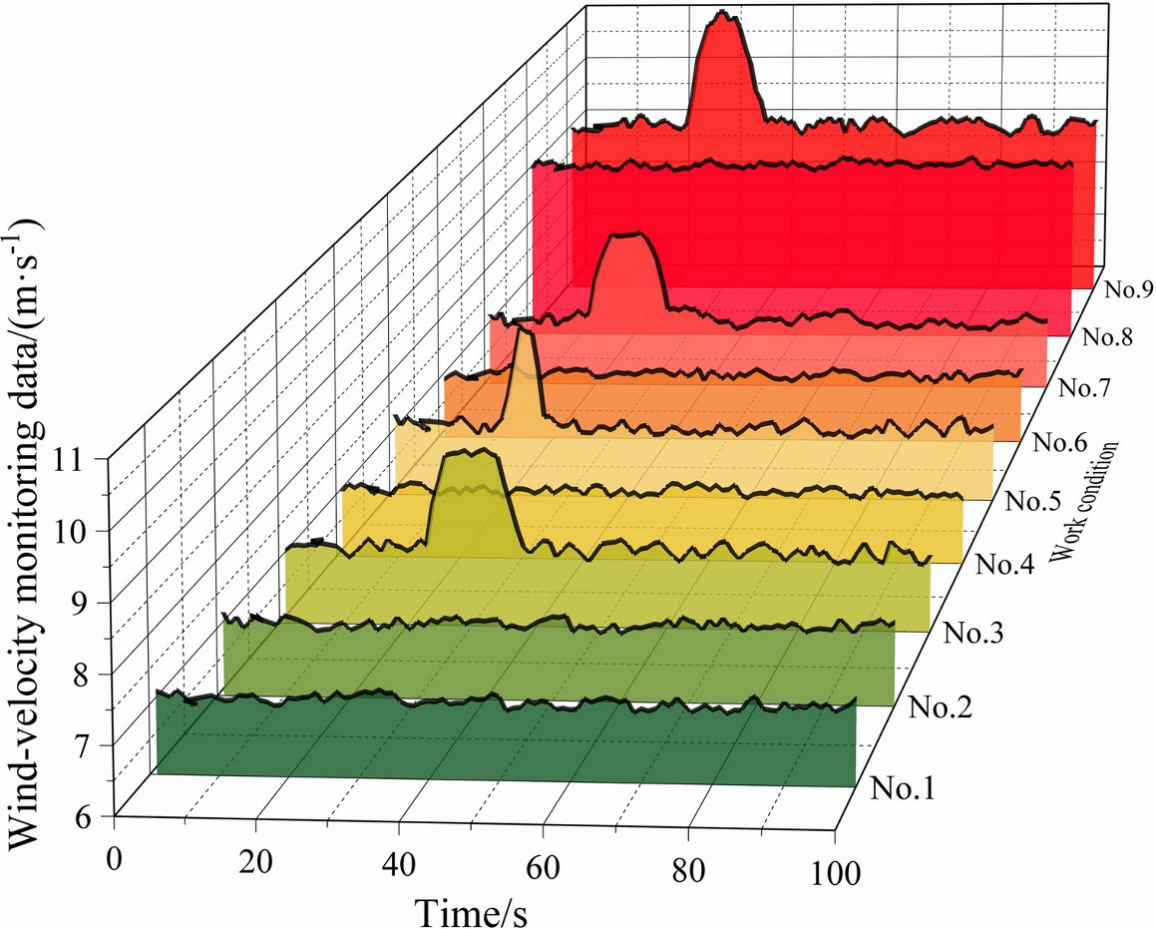
Wind-velocity data recorded at monitoring Point 2.

**Fig 12:**
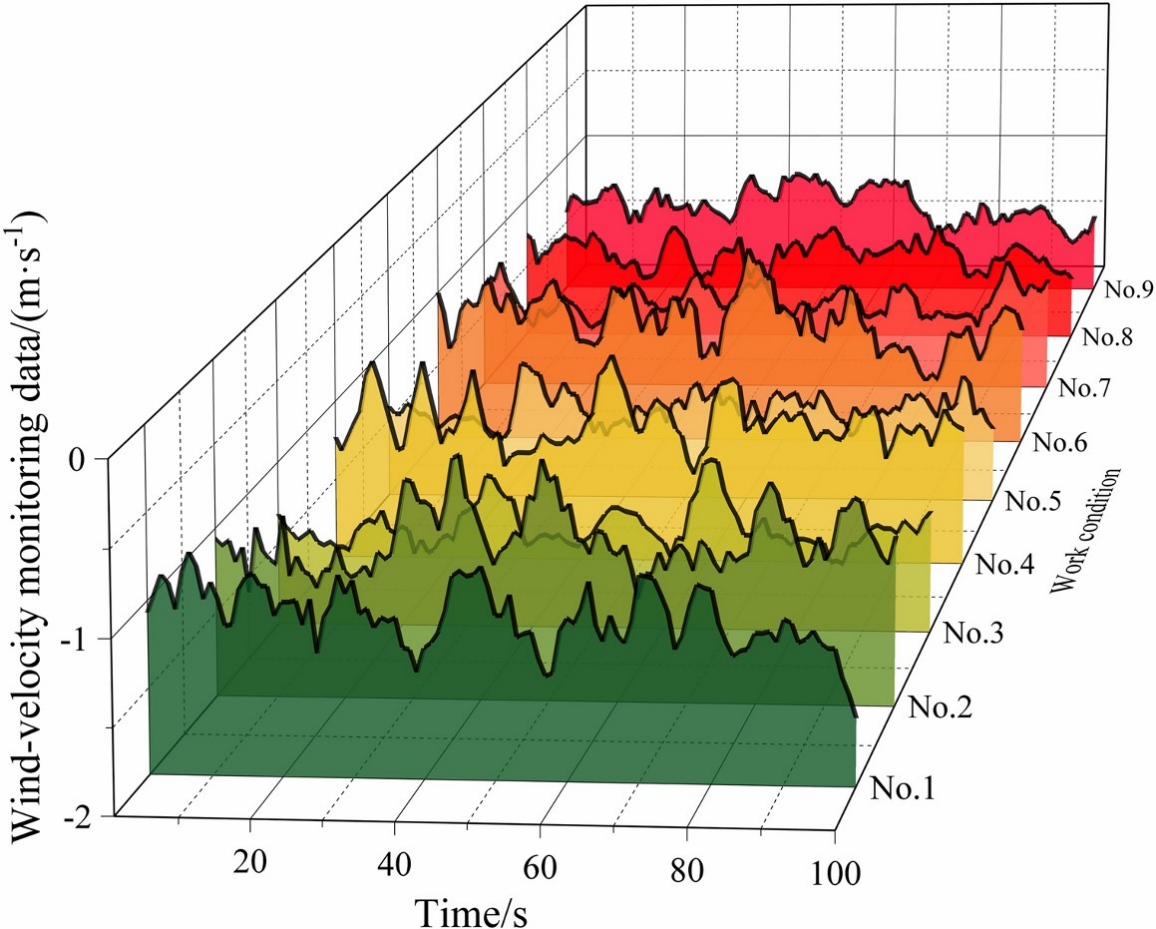
Wind-velocity data recorded at monitoring Point 3.

**Fig 13:**
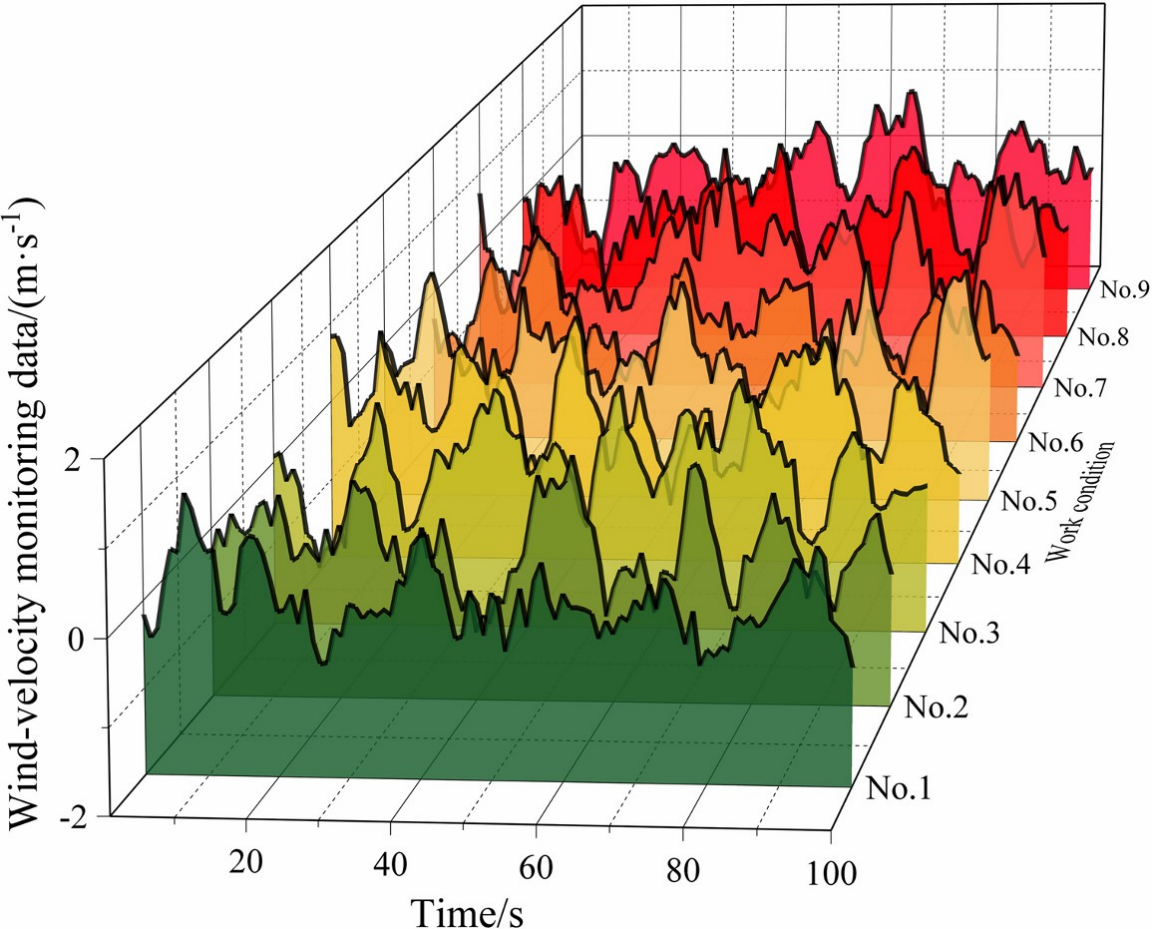
Wind-velocity data recorded at monitoring Point 4.

Table 2 lists some of the mine-car operating conditions, and Figs 10‒13 show the wind-velocity data obtained for those conditions.

**Table 2:** Mine-car operating conditions.

No.	Factor 1	Factor 2 /(m/s)	Factor 3	Factor 4 /(m/s)
**1**	No mine-car operation	/	/	8.00
**2**	A→B→A	0.06	With wind	8.00
**3**	C→D→C	0.06	With wind	8.00
**4**	A→B→A	0.10	With wind	8.00
**5**	C→D→C	0.10	With wind	8.00
**6**	A→B→A	0.06	Against wind	8.00
**7**	C→D→C	0.06	Against wind	8.00
**8**	A→B→A	0.06	With wind	10.00
**9**	C→D→C	0.06	With wind	10.00

From Table 2 and Figs 10–13, the mine-car operation generated abnormal fluctuations in the wind-velocity sensors within the tunnel containing the mine car only. When the mine car traveled through an area near a wind-velocity sensor, the corresponding wind-velocity data first rose and then fell abnormally.

## MSSW-based WPT-GBDT multi-disturbance identification method

**Fig 14:**
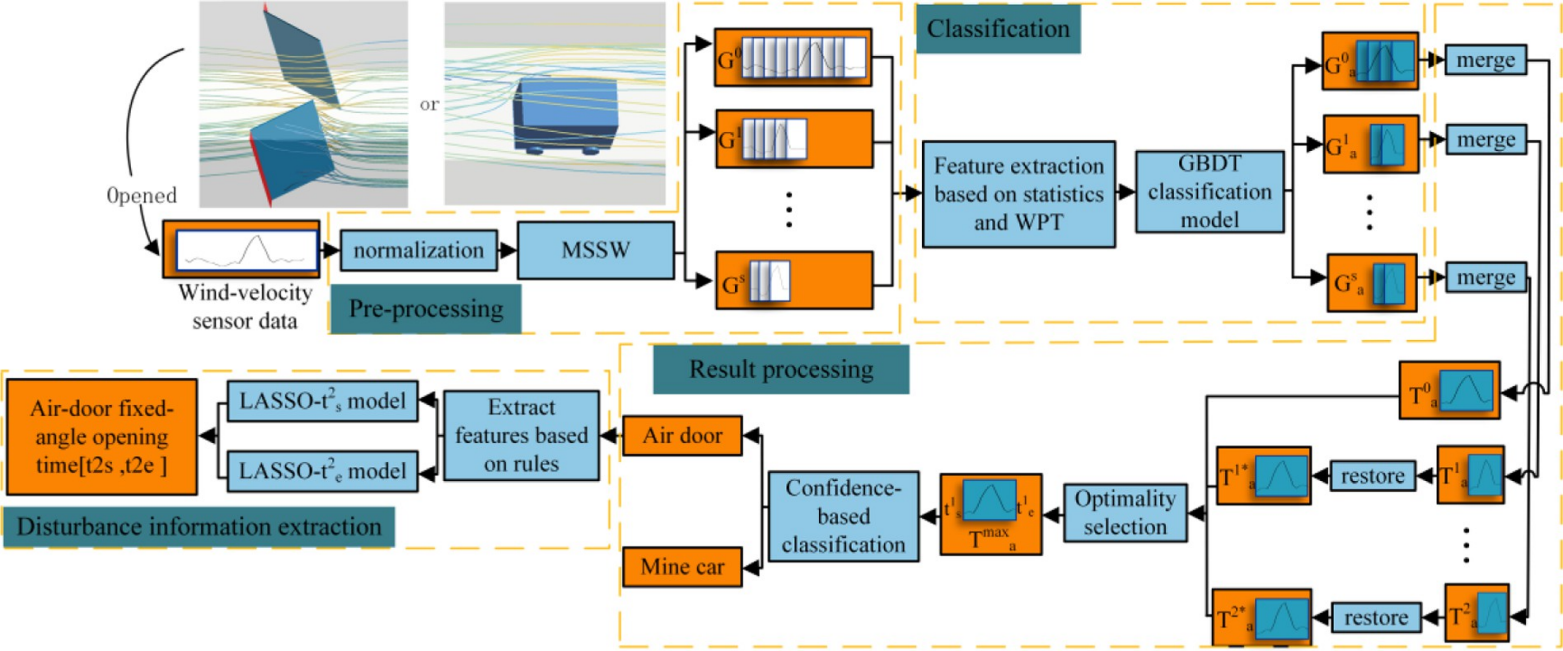
Algorithm flow chart.

Considering the two disturbance laws pertaining to the air-door and mine-car operations, an MSSW-based WPT-GBDT multi-disturbance identification algorithm is proposed in this study. The algorithm flow is shown in Fig 14. The proposed method consists of the following four steps.

1. Preprocessing: Wind-velocity sensor data segments are processed into time-series data with a varied range of [0,1] through discrete normalization. Then, MSSW is used to generate several original samples {*G*^0^, *G*^1^, …, *G*^*s*^} consisting of sub-time-series data.
2. Classification: Feature vectors are extracted from all sub-time-series data for each original sample using statistical analysis and WPT. The GBDT triple classification model is then used to classify these feature vectors. The three classification categories are normal wind-velocity data, abnormal wind-velocity fluctuation data due to air-door operation, and abnormal wind-velocity fluctuation data due to mine-car operation. Following the classification, only the sub-time-series data samples $\left\{G_{a}^{0},G_{a}^{1},\ldots ,G_{a}^{s}\right\}$ of the abnormal wind-velocity data categories are retained.
3. Result processing: First, the sub-time-series data of the abnormal wind-velocity data categories are merged according to the IoU values, with *n* time-series data samples $\left\{\{T_{0}^{0a},cd_{0}^{0a},cc_{0}^{0a}\},\{T_{1}^{1a},cd_{1}^{1a},cc_{1}^{1a}\},\ldots ,\{T_{n}^{sa},cd_{n}^{sa},cc_{n}^{sa}\}\right\}$ being generated through merging. Merged numbers from other scales are then restored to the original scale. The optimal time-series data samples $T_{a}^{\max}$ are selected using the non-maximum suppression method based on the sum of the confidence levels of all time-series data samples belonging to both abnormal fluctuation data categories. Then, the category of the anomalous fluctuation data generated by the disturbance can be determined by examining the confidence levels of the two categories. The mine wind-flow disturbance identification is then completed.
4. Disturbance information extraction: When the disturbance category is found to be abnormal wind-velocity fluctuation caused by air-door operation, the features are extracted according to the corresponding basic law of abnormal wind-velocity fluctuation. Then, two least absolute shrinkage and selection operator (LASSO) regression models are used to derive the air-door fixed-angle open time [$t_{s}^{2}$, $t_{s}^{e}$].

### Preprocessing

This section describes the normalization and discretization methods incorporated in the algorithm, and the specific changes in the preprocessing process and in the wind-velocity sensor data.

#### Normalization

Data normalization can convert wind-velocity data of different ranges into a range of [0,1] for subsequent uniform processing. In this study, deviation normalization is employed to process the wind-velocity sensor data, which is expressed as

$$x\text{'=}(x-x_{\min})/(x_{\max}-x_{\min})$$ (1)

where x' represents the normalized data; *x* represents the wind-velocity sensor data; and *x*_min_ and *x*_max_ represent the minimum and maximum wind-velocity sensor data values, respectively.

Following normalization of the *k*-th wind-velocity monitoring data point, the original dataset $\{X_{k}|x_{i}^{k}\},i=1,2,\ldots ,L$ becomes $\{X{'}_{k}|x{'}_{i}^{k}\},i=1,2,\ldots ,L$.

#### Discretization

The high time complexity of time-series data hinders further extraction of the information it contains, but discretization can effectively reduce the time complexity. Based on the MSSW method commonly used in the field of image recognition, an MSSW method for one-dimensional wind-velocity sensor data is proposed here [29, 30]. This method first scales the data according to different scales; then, a sliding window with a fixed window size and sliding distance divides the data according to the different scales.

For the method to better handle wind-velocity sensor monitoring data, the following four parameter constraints are set, in accordance with the Coal Mine Safety Regulations of China and the data characteristics:

$$Q_{min}\leq S\leq Q_{max}$$ (2)

$$Q_{max}\cdot Z\leq w$$ (3)

$$P_{min}\leq w\leq P_{max}$$ (4)

$$\left\{\begin{array}{l} {\text{t}}_{j}=\frac{w}{2}\;l_{j}>=\frac{w}{2}\\ {\text{t}}_{j}=l_{j}\;l_{j}<\frac{w}{2} \end{array}\right.$$ (5)

Eq (2) is the constraint applied to the number of scalings *S*, where *Q*_*min*_ and *Q*_*max*_ represent the minimum and maximum number of scalings, respectively. Note that *Q*_*min*_ is greater than or equal to 2. Eq (3) is the constraint on the scaling ratio and scaling times, where *Z* is the scaling ratio and *w* is the set of the sliding window scale. Eq (4) is the constraint on *w*, where *P*_*min*_ and *P*_*max*_ represent the minimum and maximum scales of a reasonable sliding window, respectively. Note that *P*_*min*_ is greater than or equal to 2, and *P*_*max*_ is less than the shortest air-door operating time. Eq (5) is the constraint on the sliding distance, where t_*j*_ is the sliding distance of the *w*-scale sliding window and *l*_*j*_ is the remaining length of the time-series data after the *w*-scale sliding window has been slid *j* times.

Considering the four constraints, we set *S* to 4, *Z* to 2, and *w* to 4. When these parameters are used, the *k*-th sample of the normalized wind-velocity sensor data $\{X{'}_{k}|x{'}_{i}^{k}\},i=1,2,\ldots ,L$ is discretized through a multi-scale sliding window to generate multiple sub-time-series data samples, and each time-series data sample is expressed as follows:

$$\left\{\begin{array}{l} T_{k,j}^{s}=\{x_{2j-1}^{s},x^{s}{}_{2j},x^{s}{}_{2j+1},x^{s}{}_{2j+2}\}\;2j+2\leq L^{s}\\ T_{k,j}^{s}=\{x_{L^{s}-3}^{s},x^{s}{}_{L^{s}-2},x^{s}{}_{L^{s}-1},x^{s}{}_{L^{s}}\}\;2j+2>L^{s} \end{array}\right.$$ (6)

where $T_{k,j}^{s}$ represents the sub-time-series data sample in the *j*-th sliding window after division by the *k*-th sample of wind-velocity sensor data *s* times; $x_{2j-1}^{s},x^{s}{}_{2j},x^{s}{}_{2j+1},x^{s}{}_{2j+2}$ represent the data at times 2*j*-1, 2*j*, 2*j*+1, 2*j*+2; and $x_{L^{s}-3}^{s},x^{s}{}_{L^{s}-2},x^{s}{}_{L^{s}-1},x^{s}{}_{L^{s}}$ represent the data at times *L*^*s*^-3, *L*^*s*^-2, *L*^*s*^-1, *L*^*s*^.

**Fig 15:**
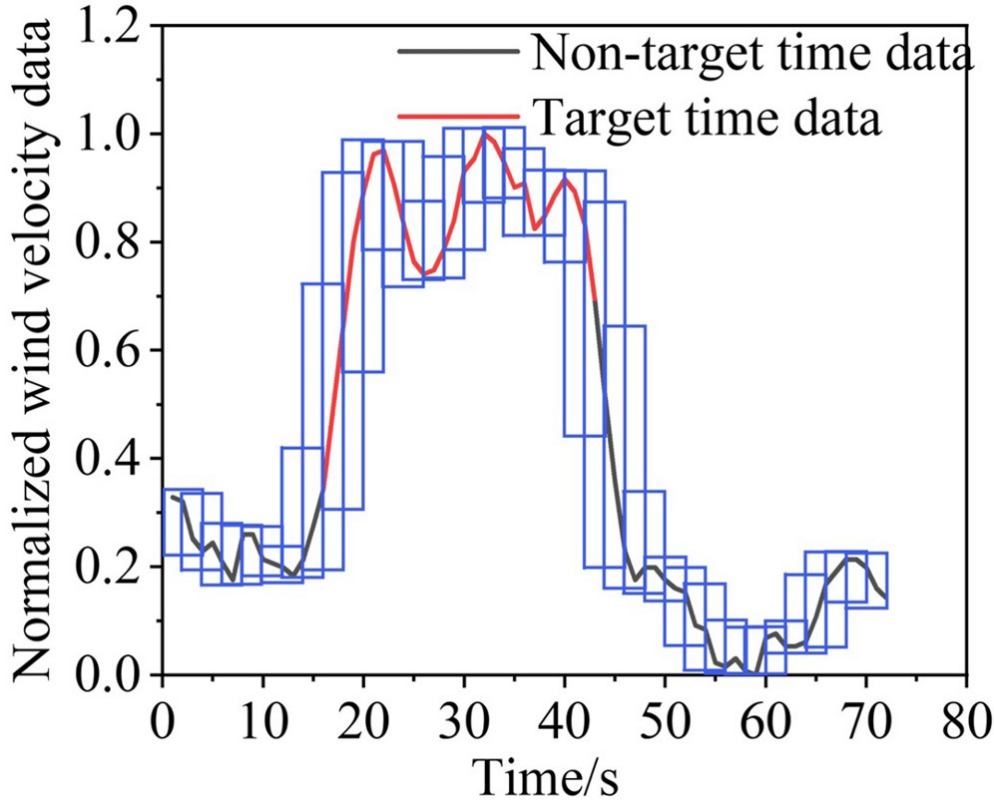
Sliding window discretized with 0 scalings.

**Fig 16:**
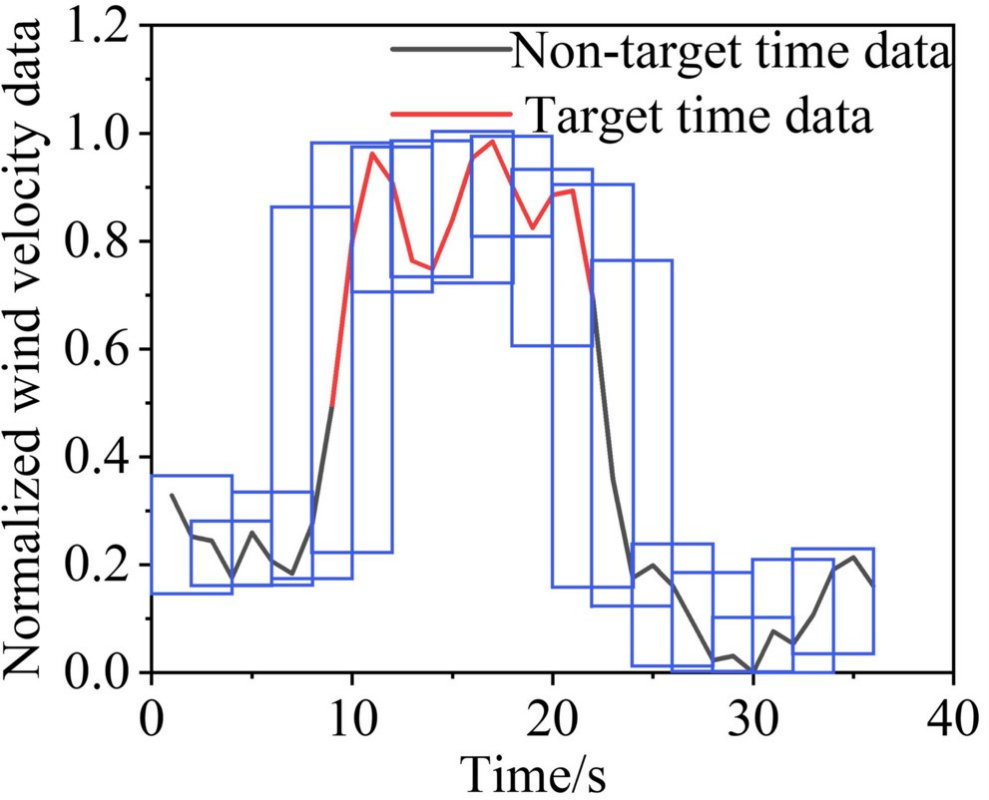
Sliding window discretized with 1 scaling.

**Fig 17:**
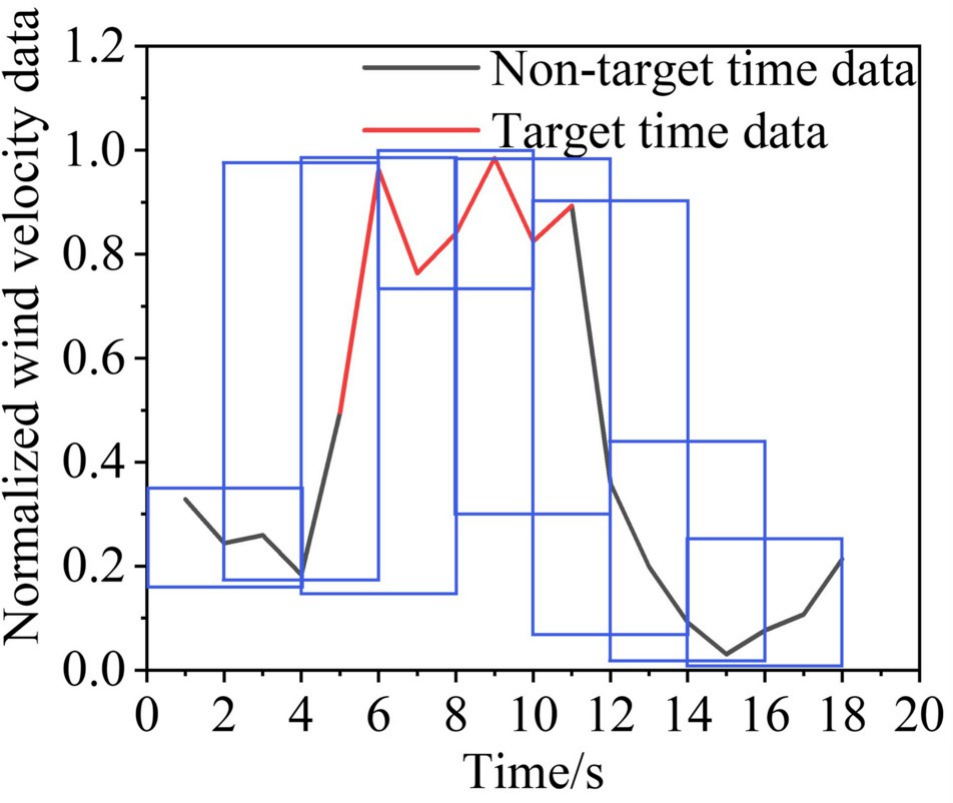
Sliding window discretized with 2 scalings.

**Fig 18:**
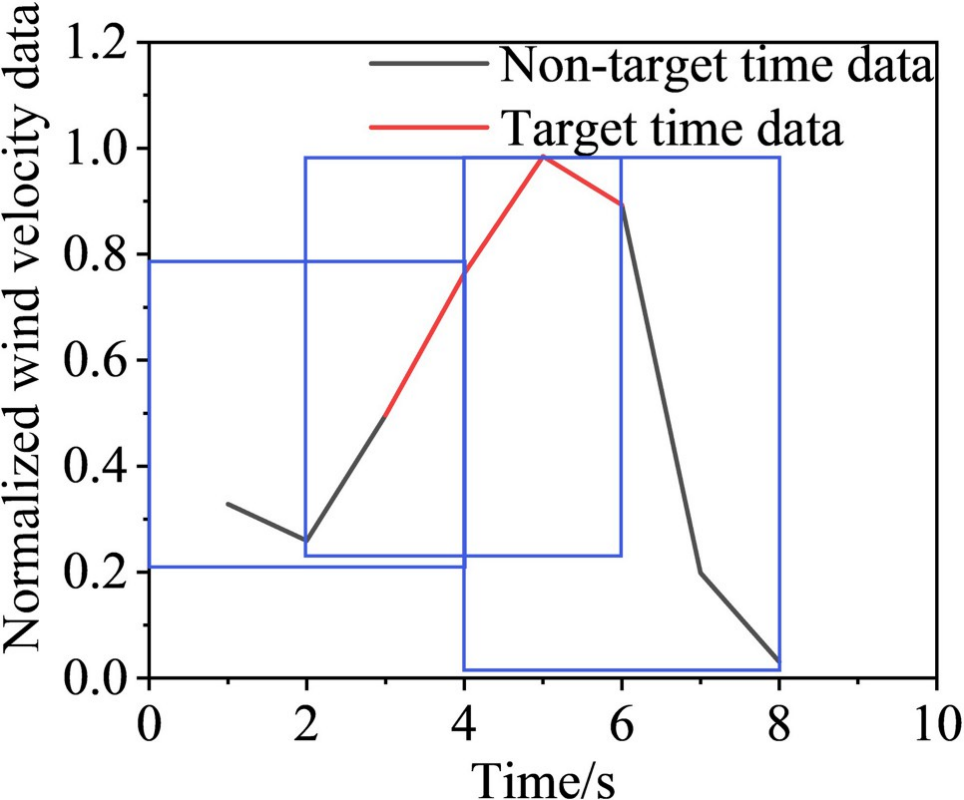
Sliding window discretized with 3 scalings.

Following the sliding window discretization of the data at all scales, multiple raw samples {*G*^0^, *G*^1^, *G*^2^, *G*^3^} are generated, each of which is composed of sub-time-series data discretized from the data at the corresponding scale. To better describe this process, we take a segment of wind-velocity sensor data as an example and show the discretization process for *S* = 0, 1, 2 and 3; the results are in Figs 15,16,17, and 18, respectively.

### Classification

The classification step consists of two parts: sub-time-series feature extraction and use of the GBDT model to classify the sub-time series.

#### Feature extraction based on statistics and WPT

Statistical features can fully express the global information of sub-time-series data. The statistical features used in this study were the mean $\bar{x}$, minimum *x*_min_, maximum *x*_max_, and variance *σ*^2^ of each sub-time-series, which were calculated as follows:

$$\bar{x}=\sum_{i}^{k}x_{i}/k$$ (7)

$$x_{min}=\min(T_{k,j}^{s})$$ (8)

$$x_{\max}=\max(T_{k,j}^{s})$$ (9)

$$\sigma^{2}=\sum_{i}^{k}\left(x_{i}-\bar{x}\right)/k$$ (10)

Following extraction of the global information, a proposed WPT-based fluctuation feature extraction method is implemented to better represent the fluctuation information embedded in the sub-time-series data.

In this approach, WPT is further developed on the basis of WT. The method through which the data frequency band is divided equally improves the poor resolution of the WT in the high-frequency signal band, enabling finer and more comprehensive data analysis. The data decomposition process implemented via the WPT method is expressed as

$$\left\{\begin{array}{l} a_{2j}(n)=\sqrt{2}\sum_{k}^{K-1}a_{j}(2n-k)h_{1}(k)\\ a_{2j+1}(n)=\sqrt{2}\sum_{k}^{K-1}a_{j}(2n-k)h_{0}(k) \end{array}\right.$$ (11)

where *h*_0_(*k*) and *h*_1_(*k*) are low- and high-pass filters, respectively; and *K* is the filter length.

The wavelet basis used here is the WPT of the db1 function for the sub-time-series data. As each sub-time-series data sample has length 4, it is decomposed into, at most, two layers, and each layer consists of high- and low-frequency coefficients. The entropy sums of the high- and low-frequency coefficients obtained from the multilayer decomposition are calculated to obtain the fluctuation characteristics, as follows:

$$\left\{\begin{array}{l} e_{js}=\sum_{i=1}^{n_{s}}-cs_{ji}\times \log_{2}^{cs_{ji}}\\ e_{jd}=\sum_{i=1}^{n_{d}}-cd_{ji}\times \log_{2}^{cd_{ji}} \end{array}\right.$$ (12)

where *e*_*js*_ and *e*_*jd*_ represent the entropy sums of the low- and high-frequency coefficients in layer *j*, respectively; *cs*_*ji*_ and *cd*_*ji*_ represent the *i*-th low- and high-frequency coefficients in layer *j*, respectively; and *n*_*s*_ and *n*_*d*_ represent the total numbers of low- and high-frequency coefficients in layer *j*, respectively.

According to Eq 12, four fluctuation features are extracted by WPT: *e*_1*s*_ and *e*_1*d*_, which correspond to the total entropy of the first layer, incorporating the low- and high-frequency coefficient entropy, respectively; and *e*_2*s*_ and *e*_2*d*_, which correspond to the total entropy of the second layer, incorporating the low- and high-frequency coefficient entropy, respectively.

In summary, the feature vector $T_{k,j}^{s}$ extracted from each sub-time-series data sample consists of four statistical features and four volatility features, such that

$$C_{k,j}^{s}={\left(\bar{x'},x{'}_{min},x{'}_{max},\sigma^{2},e_{1s},e_{1d},\ldots ,e_{Ls},e_{Ld}\right)}^{T}$$ (13)

#### GBDT model classification

**Fig 19:**
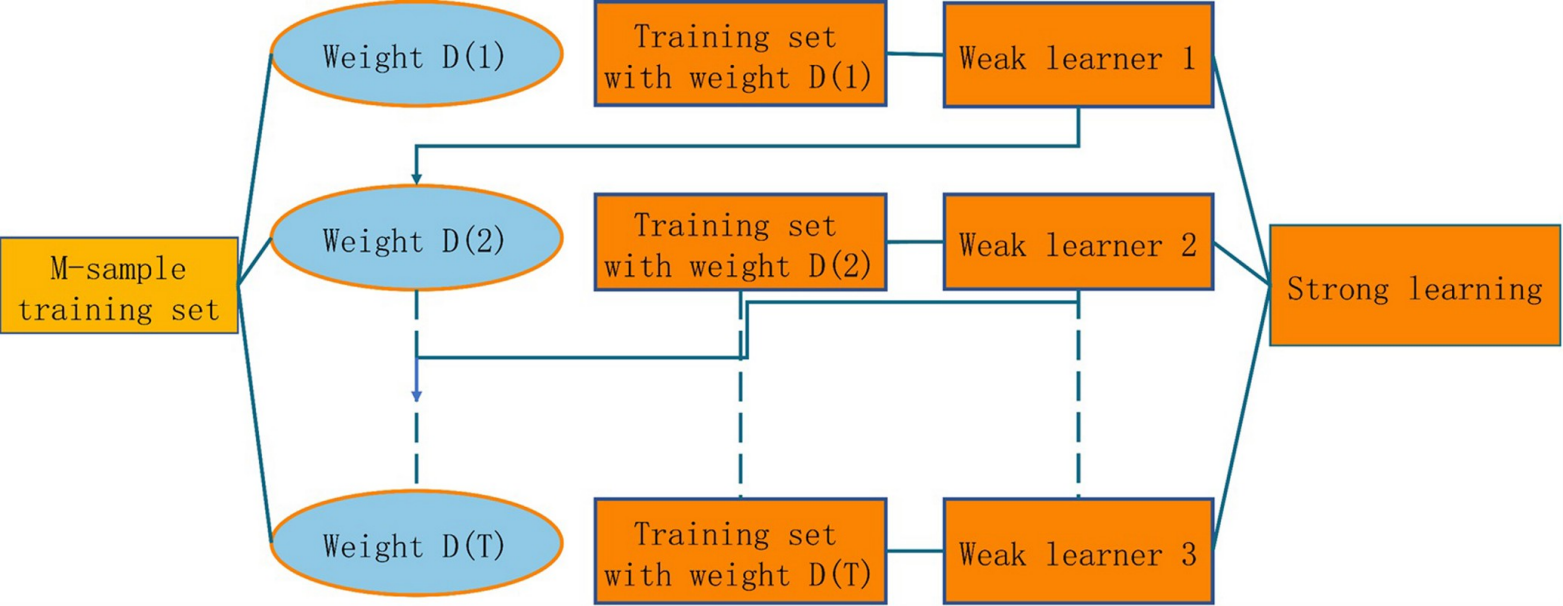
Boosting algorithm principle.

We use GBDT as the algorithm classification model. GBDT is an effective combination of decision trees and integration ideas achieved by constructing a weak set of learners (trees) and accumulating the results of multiple decision trees as the final prediction output. The underlying concept is the boosting method; i.e., the base classifiers are stacked in layers and, in each layer, higher weights are given to samples incorrectly classified by the previous layer of base classifiers during training. In testing, the final result is obtained based on the weighting of the results of each layer of classifiers, as shown in Fig 19.

The GBDT model is used to triple-classify the sub-time-series data according to the disturbance categories of normal, air-door operation, and mine-car operation. The air-door operation category incorporates wind-velocity data obtained in the time period from the air-door opening to closing, and the mine-car operation category refers to wind-velocity data obtained when the mine-car is 200 mm from the windward side of the wind-velocity sensor to 700 mm from the ventilation side of the sensor. After the classification is completed, only sub-time-series data of category $T_{k,j}^{sa}$ for air-door and mine-car operations are retained. The sub-time-series data belonging to the air-door and mine-car operation categories have confidence levels of $cd_{k,j}^{sa}$ and $cc_{k,j}^{sa}$, respectively; that is, the sub-time-series data belonging to the air-door and mine-car operation categories are selected from the original sample {*G*^0^, *G*^1^,…,*G*^*s*^}, which contains the sub-time-series data of all categories, considering the confidence levels of $\left\{T_{k,j}^{sa},cd_{k,j}^{sa},cc_{k,j}^{sa}\right\}$for these two categories. Then, multiple data samples $\left\{G_{a}^{0},G_{a}^{1},\ldots ,G_{a}^{s}\right\}$are formed.

### Classification result processing

In this study, two processing methods were employed for the classification results: merge & restore and sorting & judgment. These methods were selected for the reasons given below.

1. Merge & restore: The sub-time-series data of the abnormal wind-velocity fluctuation data category generated by the air-door or mine-car operation are screened through GBDT. These sub-time-series data are still discrete, whereas the abnormal wind-velocity fluctuation data generated by the air-door and mine-car operations are continuous; thus, the sub-time-series data of each sample should be subjected to a sequence based on the IoU values of the sub-time-series data, IoU value refers to the overlap between two time series. The merged parts of the time-series data should be downscaled to obtain the real data because they have undergone scaling during discretization.
2. Sorting & judgment: After the time-series data are reduced to the data before scaling, they should be grouped and sorted according to their IoU values and confidence degree, because of overlapping between them. Once the optimal time-series data have been obtained through sorting, they belong to one of the two abnormal disturbance categories; thus, the cause of the abnormal disturbance is determined by categorizing the sub-time-series data according to the confidence degree that they belong to the air-door or mine-car operation category.

#### Merge & restore

As the scales of the different samples vary, only sub-time-series data within a single sample are considered for series merging. The combination of two sub-time-series data elements is determined based on whether an overlap exists between them.

An overlap of two sub-time-series data elements is indicated by an IoU value between them that exceeds 0. The IoU value is calculated as follows:

$$IoU_{ab}=\frac{T_{k,a}^{s}\cap T_{k,b}^{s}}{T_{k,a}^{s}\cup T_{k,b}^{s}}$$ (14)

where $T_{k,a}^{s}$ and $T_{k,b}^{s}$ represent two sub-time-series data elements.

The joint method is used to merge the two sub-time-series data elements. The combination of two overlapping sub-time-series data elements and the confidence calculation of the merged sub-time-series data elements is expressed as

$$\left\{\begin{array}{l} T_{k,m}^{sa}=T_{k,a}^{sa}\cap T_{k,b}^{sa}\\ cd_{k,m}^{sa}=\frac{cd_{k,a}^{sa}+cd_{k,b}^{sa}}{2}\\ cc_{k,m}^{sa}=\frac{cc_{k,a}^{sa}+cc_{k,b}^{sa}}{2} \end{array}\right.$$ (15)

where $T_{k,m}^{sa}$ represents the merged sub-time-series data and $cd_{k,m}^{sa}$ and $cc_{k,m}^{sa}$ represent the confidence degrees that the merged sub-time-series data belong to the air-door and mine-car operation categories, respectively.

Eqs (14) and (15) are used to complete the merging of all sub-time-series data in the data sample. Once *n* $\left\{\{T_{0}^{0a},cd_{0}^{0},cc_{0}^{0}\},\{T_{1}^{1a},cd_{1}^{1a},cc_{1}^{1a}\},\ldots ,\{T_{n}^{sa},cd_{n}^{sa},cc_{n}^{sa}\}\right\}$ is obtained, the time-series data obtained by synthesizing the sub-time-series data for one of the scaling scales is restored to 0-scale scaling.

#### Sorting & judgment

**Fig 20:**
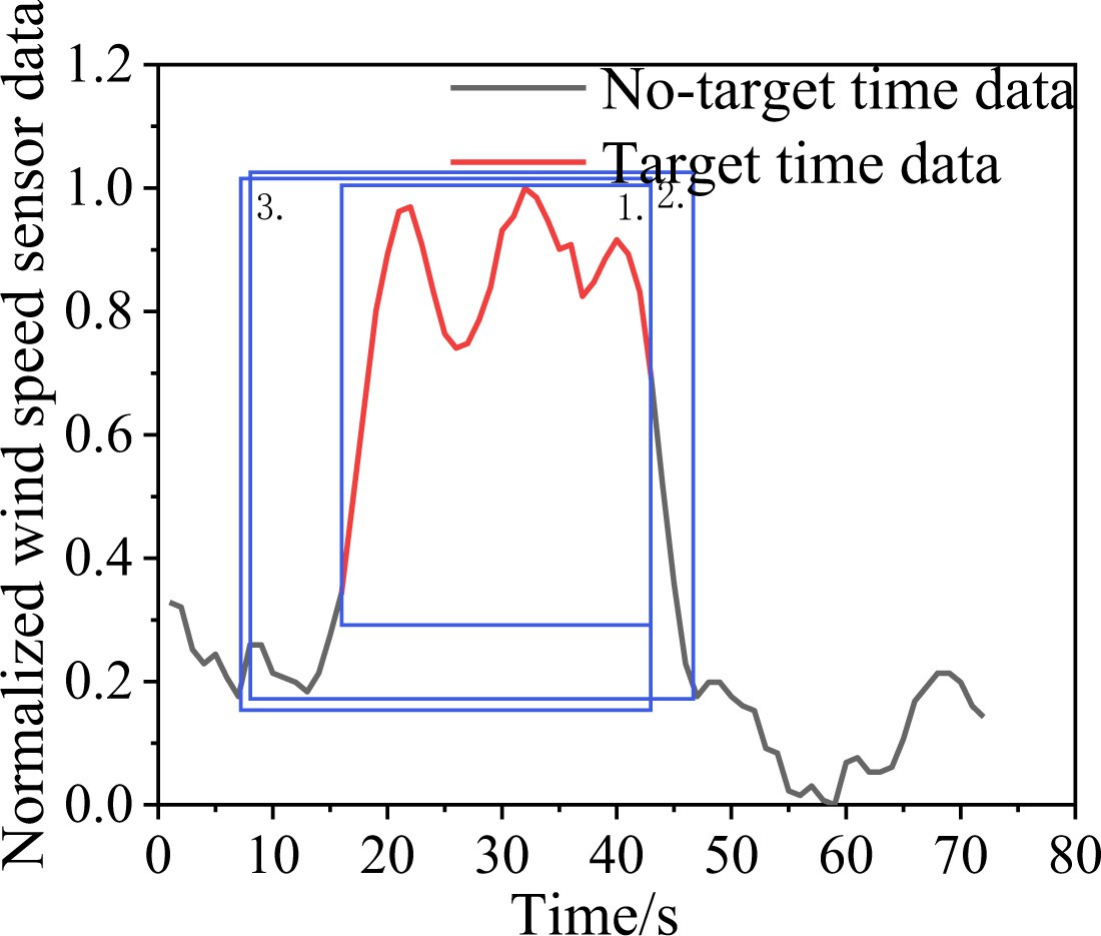
Initial identification results with overlapping.

Once all time-series data are restored, they constitute the preliminary algorithm disturbance recognition results, which overlap as shown in Fig 20.

As apparent from Fig 20, the algorithm gives several preliminary recognition results for a perturbation. To select the optimal recognition result, the preliminary recognition results for different perturbations are divided into different groups according to the IoU threshold. In the same group, the best recognition result and the perturbation class of the recognition result are selected according to the confidence level using non-maximal value suppression. The specific implementation process is detailed in **Algorithm 1**.

#### Algorithm 1. Sorting judgment

Input: preliminary result $\left\{\{T_{0}^{0a},cd_{0}^{0},cc_{0}^{0}\},\{T_{1}^{1a},cd_{1}^{1a},cc_{1}^{1a}\},\ldots ,\{T_{n}^{sa},cd_{n}^{sa},cc_{n}^{sa}\}\right\}$

Output: identication result $O=\{T_{0}^{O}=\{T_{0}^{0\text{o}},cd_{0}^{0\text{o}}\},\ldots ,T_{z}^{O}=\{T_{z}^{\text{zo}},cc_{z}^{\text{zo}}\}\}$

1. *O = {}*
2. for x in 0, …, s do
3. d = 0
4. $T_{d}^{0\text{o}}=\{\}$
5. for y in 0, …, s do
6. calculate *IoU*_*x*,*x*+1_ by Eq. ( 10 )
7. if *IoU*_*x*,*x*+1_ > 0.5 then
8. $T_{0}^{O}$ append $\left\{T_{0}^{0a},cd_{0}^{0},cc_{0}^{0}\right\}$)
9. end if
10. if *IoU*_*x*,*x*+1_ ≤ 0.5 then
11. *O* append($T_{0}^{O}$)
11. d = d+1
10. end if
11. for T in *O* do
12. num_list = [0,…len(T)]
13. sort from largest to smallest by (max(T[num_list[0]][1], T[num_list[0]][2]),…, max(T[num_list[len(T)]][1], T[num_list[len(T)]][2]))
14. for T in *O* do
15. T = T[0]
16. T = {T[0],max(T[1],T[2])}

#### Disturbance information extraction

When the identified time-series data perturbation category is air-door operation, the LASSO regression model is used to further extract air-door operation information. LASSO regression, also known as “minimum absolute shrinkage and selection operator regression,” is a multiple linear regression technique with an additional penalty parametric (L1) to enhance model stability and prevent overfitting by ignoring useless features. The minimization objective function equation is

$$Q(\beta )=||\text{y}-X\beta |{|}^{2}+||\beta |{|}_{1}$$ (16)

where *Q*(*β*) minimizes the objective function; *X* is the design matrix; *β* is the unknown parameter vector; *y* is the linear regression equation; ||*β*||_1_ is the L1 norm, and the specific expression formula is $\sum_{i=1}^{n}\left|\beta_{i}\right|$.

Two LASSO regression models are established in this method: a model with the start time of the optimal damper fixed-angle opening time-series as the regression target, and a model with its end time modification as the regression target. The feature vector used for LASSO model training is

$$C_{l}={\left(t_{s},t_{e},T,cd,T_{\max},T_{\min},T_{\sigma },\bar{T}\right)}^{T}$$ (17)

where *C*_*l*_ is the set of features of the two regression models; *t*_*s*_, *t*_*e*_, *T*, and *cd* are the start and end times, the duration, and the confidence level of the air-door operation time series; and *T*_min_, *T*_max_, *T*_*σ*_, and $\bar{T}$ are the minimum value, maximum value, standard deviation, and mean of the air-door operation time-series data, respectively.

Following derivation of the two time corrections, we obtain the air-door fixed-angle opening time and the wind-velocity data during this time.

## Results and discussion

### Dataset and evaluation indicators

The recognition performance of the proposed method for various types of disturbances was verified through comparisons with other data sets and algorithms. The compositions and disturbance category divisions of the other data sets used for performance verification of the proposed algorithm are listed in Table 3.

**Table 3:** Dataset composition.

Disturbance category	Data contained	Classification model training	Regression model training	Overall method testing
**Air-door operation**	120 wind-velocity data segments	50%	30%	20%
**Mine-car operation**	100 wind-velocity data segments	70%	/	30%

Following dataset preparation, three evaluation indicators were selected: accuracy (AC), precision (PR), and recall (RE). These indicators were used to evaluate the algorithm disturbance recognition performance, and were calculated based on the true positive samples (TP) correctly identified by the method as positive class; the true negative samples (TN) correctly identified by the method as negative class; the false negative samples (FN), i.e., positive samples incorrectly identified by the method as negative class; and the false positive samples (FP), i.e., negative samples incorrectly identified by the method as positive class.


$$AC=\frac{TP+TN}{TP+FP+TN+FN}$$ (18)

$$PR=\frac{TP}{FP+TP}$$ (19)

$$SE=\frac{TP}{TP+FN}$$ (20)

### Algorithm comparison

**Fig 21:**
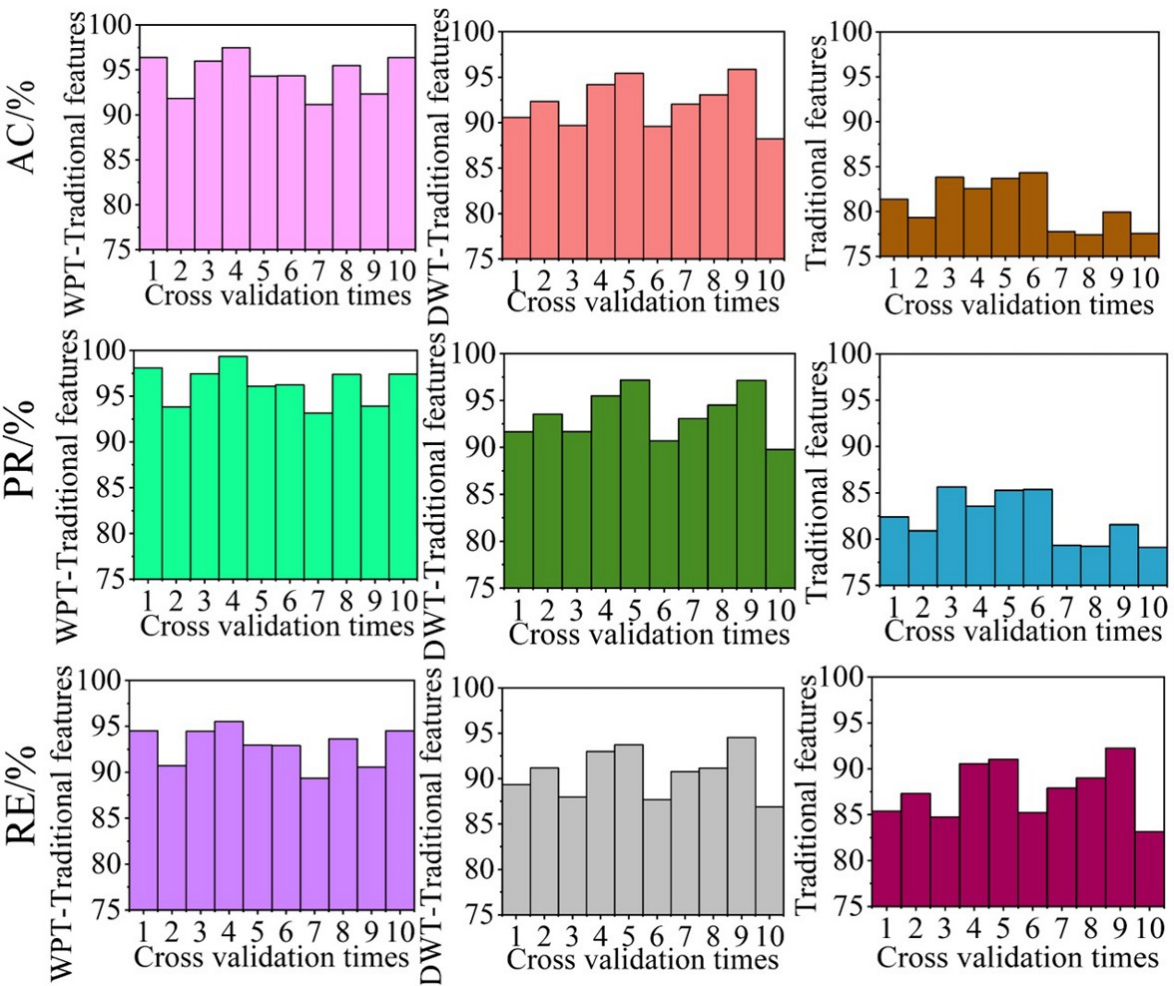
Performance comparison of disturbance recognition algorithms employing different feature extraction methods.

GBDT with a maximum number of iterations of 100, a learning rate of 1, and a loss function of pair likelihood loss function was selected as the classification model and the disturbance recognition performances of algorithms using WPT and statistical features, DWT and statistical features, and statistical features only were compared. To avoid contingency, the performances of each method were cross-verified 10 times. The evaluation indicator results for each cross-validated algorithm are shown in Fig 21, and the average values of the disturbance recognition performance indicators for the *k*-fold cross-validated algorithm (*k* = 10) are listed in Table 4.

**Table 4:** Average performance comparison of disturbance recognition algorithms employing different feature extraction methods.

Index	WPT- statistical features /%	DWT- statistical features /%	Statistical features /%
**AC**	94.58	92.10	82.95
**PR**	95.70	92.31	86.85
**RE**	92.99	90.52	81.21

**Fig 22:**
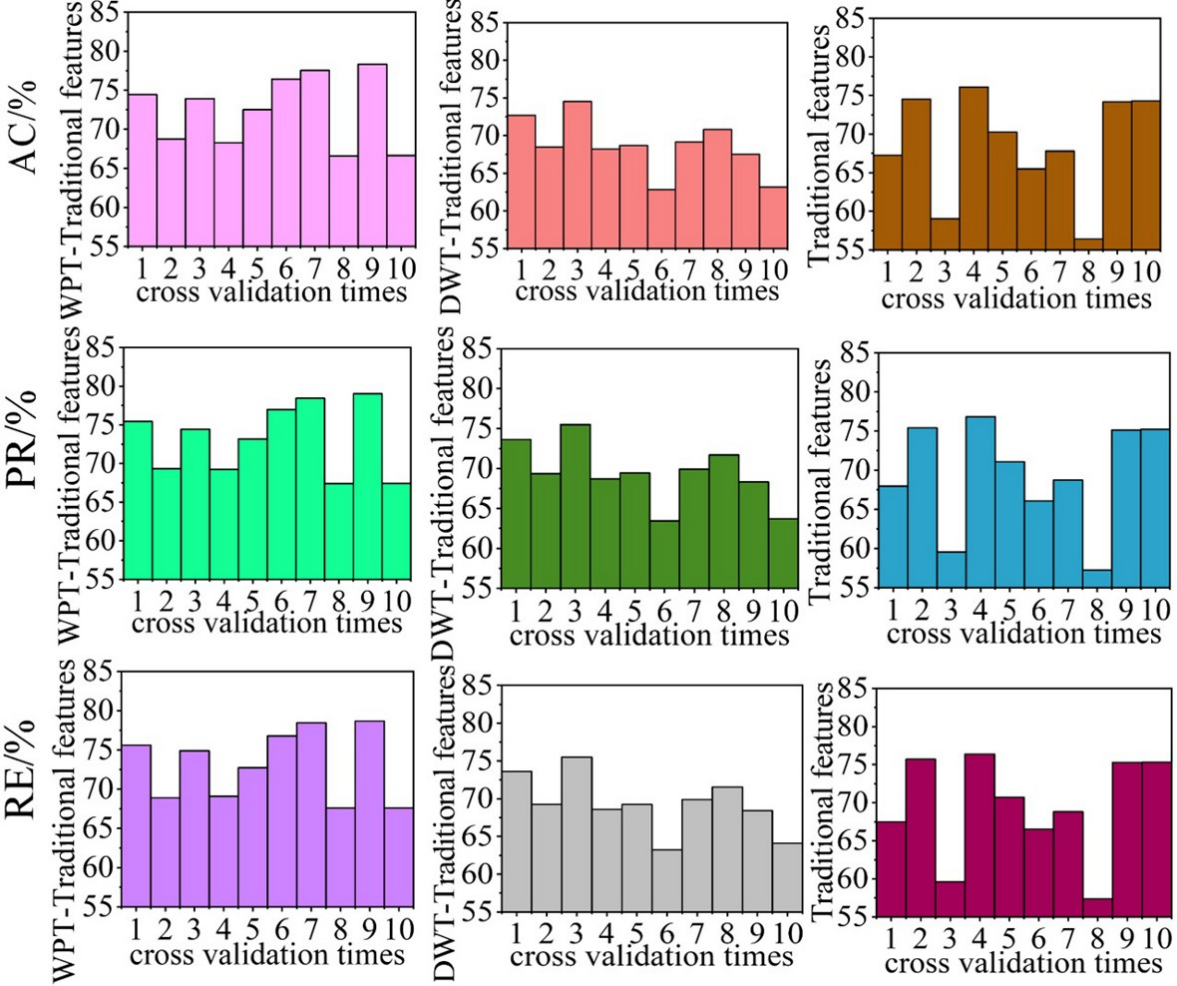
Disturbance information extraction performance comparison for algorithms using different feature extraction methods.

The perturbation extraction performances of the same algorithms were compared, as shown in Fig 22. The average evaluation results for the 10-times cross-validated algorithms are listed in Table 5.

**Table 5:** Disturbance information extraction performance comparison for algorithms using different feature extraction methods.

Index	WPT- statistical features /%	DWT- statistical features /%	Statistical features /%
**AC**	72.36	68.60	68.54
**PR**	73.08	69.38	69.32
**RE**	71.02	67.34	67.32
**comparison**	Best	Better	Worse

From Figs 21 and 22 and Tables 4 and 5, the algorithm using the DWT-statistical feature method for feature extraction for disturbance identification exhibited superior performance than that using only the statistical feature method (Table 4); that is, the AC, PR, and RE of the former were 9.15%, 5.46%, and 9.31% higher, respectively; However, the algorithm using the DWT-statistical feature method exhibited similar performance to that using only the statistical feature method in the disturbance information extraction task (Table 5), with the AC, PR, and RE values of the former being only 0.06%, 0.06%, and 0.02% higher, respectively. For the disturbance identification task (Table 4), the algorithm using the WPT-statistical feature method for feature extraction exhibited superior performance to that using the DWT-statistical feature method for feature extraction; the AC, PR, and RE values of the former were 2.48%, 3.39%, and 2.47% higher, respectively. As regards disturbance information extraction, the algorithm using the WPT-statistical feature method for feature extraction also exhibited superior performance to that using the DWT-statistical feature method, with AC, PR, and RE results that were 3.76%, 3.7%, and 3.68% higher, respectively. Thus, the algorithm with superior feature extraction performance also exhibits better perturbation information extraction performance.

In summary, among the three feature extraction methods, the algorithm using WPT-statistical features for feature extraction has superior performance to that using DWT-statistical features for feature extraction, which in turn performs better than that using only statistical features for feature extraction.

### Variation of classification model

**Fig 23:**
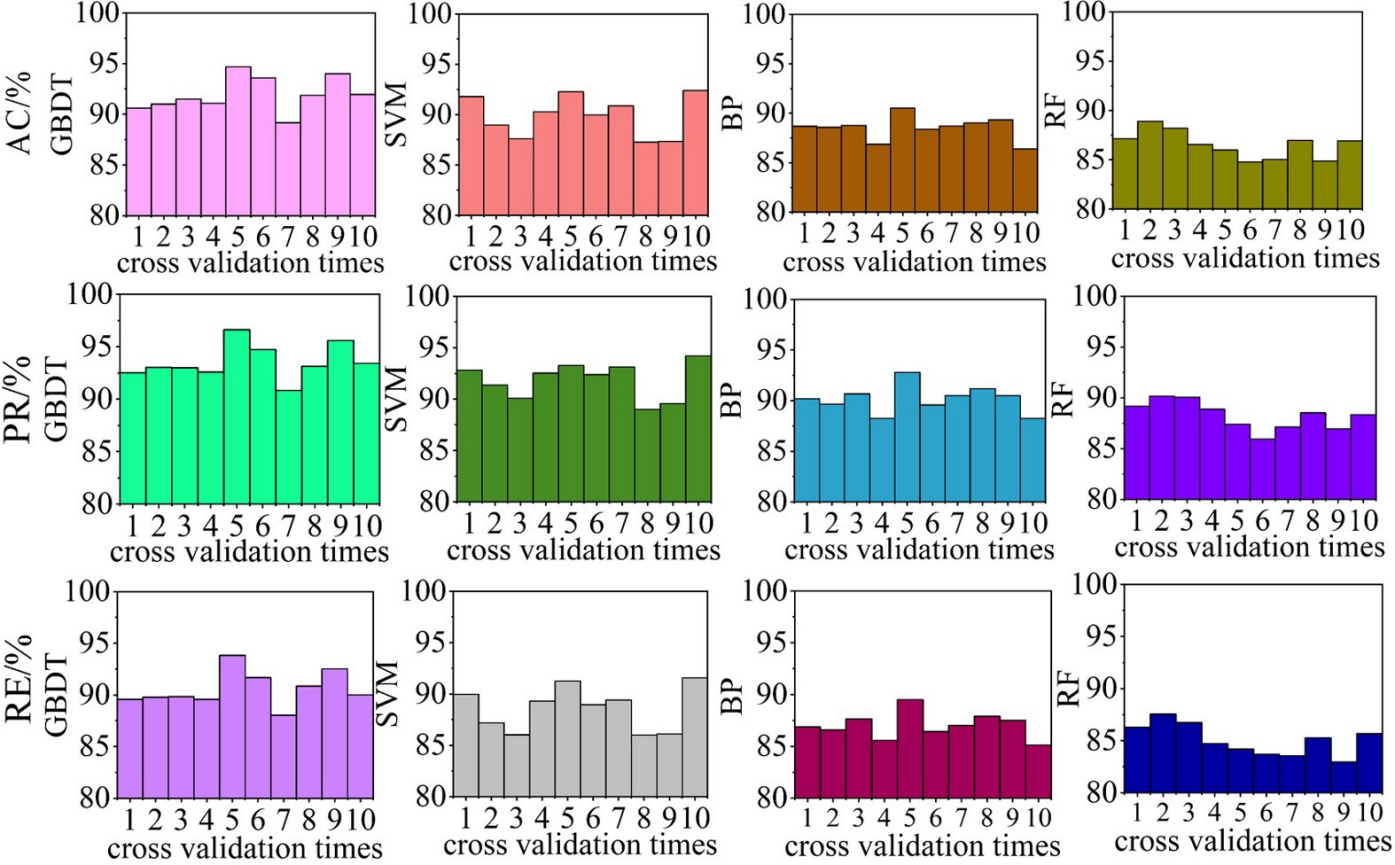
Disturbance recognition performance comparison for algorithms with different classification models.

To explore the advantages of GBDT as a classification model in the proposed algorithm, algorithms using SVM, Back Propagation Neural Network, and RF as classification models were examined for comparison. To eliminate chance, the performance of each algorithm was cross-validated 10 times. The perturbation recognition performance of each cross-validated algorithm is shown in Fig 23, and the average evaluation indicator values for the perturbation recognition performance of the 10-fold cross-validated algorithms are listed in Table 6.

**Table 6:** Average disturbance recognition performance comparison for algorithms with different classification models.

Index	GBDT/%	SVM/%	BP/%	RF/%
**AC**	94.58	89.87	88.527	86.533
**PR**	95.70	91.829	90.158	88.248
**RE**	92.99	88.578	87.018	85.063

**Fig 24:**
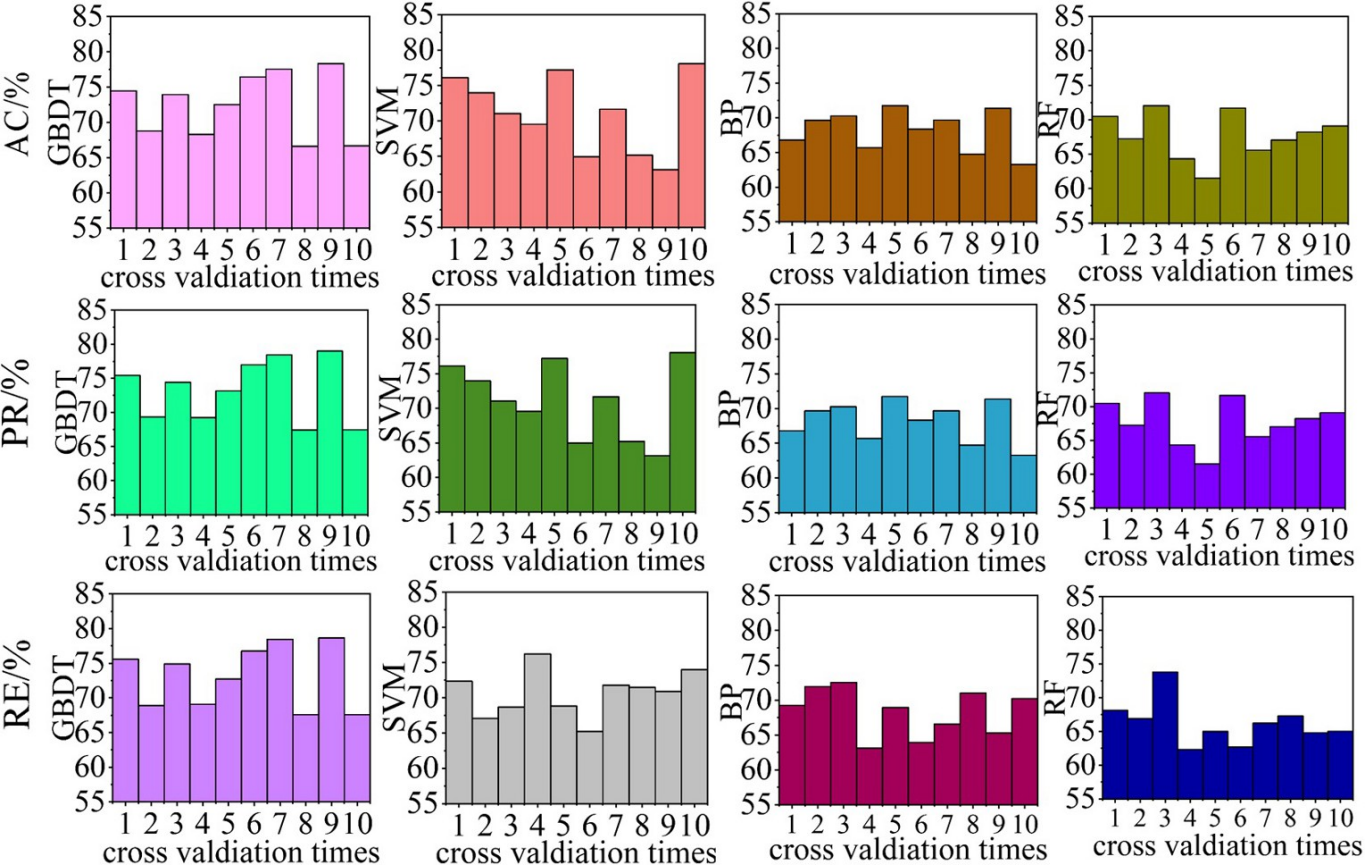
Disturbance information extraction performance comparison for algorithms with different classification models.

The perturbation information extraction performance of each 10-fold cross-validated algorithm is shown in Fig 24, and the average evaluation indicator results are listed in Table 7.

**Table 7:** Disturbance information extraction performance comparison for algorithms with different classification models.

Index	GBDT/%	SVM/%	BP/%	RF/%
**AC**	72.35	68.89	66.71	66.87
**PR**	73.08	71.09	68.15	67.72
**RE**	71.02	70.65	68.28	66.22
**comparison**	Best	Better	Worse	Worst

From Figs 23 and 24 and Tables 6 and 7, the algorithm with the RF classification model exhibited the worst performance, with AC, PR, and RE values for perturbation recognition of less than 90%, and AC, PR, and RE values for perturbation information extraction of less than 70%. When BP was used as the classification model, the algorithm exhibited slightly better performance than that with the RF classification model; however, the AC, PR, and SE values for the perturbation information extraction task were less than 70%. When SVM was used as the classification model, the algorithm performance was second to that with the GBDT classification model; the AC, PR, and SE values for the perturbation recognition task exceeded 90%; however, for the perturbation information extraction task, only the PR and SE exceeded 70%, with the AC being lower. The proposed algorithm with GBDT as the classification model yielded the best results, with the AC, PR, and RE results for the perturbation recognition task all exceeding 90%, and the AC, PR, and RE values for the perturbation information extraction task all exceeding 70%. In summary, the performances of the four classification models can be ranked as follows: GBDT > SVM > BP > RF.

## Conclusions

An MSSW-based WPT-GBDT mine multi-disturbance identification algorithm was proposed in this study, and was designed to identify the causes of abnormal fluctuations in mine wind-velocity sensors (air-door or mine-car operation) from abnormal fluctuations in the wind-velocity sensor data; the proposed algorithm also further extracts disturbance information after identification. The proposed method was verified through similarity experiments of air-door and mine-car operations, and the following conclusions were drawn.

1. In the proposed method, WPT extracts the hidden features of the wind-velocity data. The classification model is GBDT, which has high average recognition accuracy and generalization performance for recognition and classification of disturbances such as air-door or mine-car operation; thus, the probability of false sensor alarms due to air-door and mine-car operation is reduced.
2. Comparison of the performances of algorithms using other feature extraction methods with the proposed algorithm using WPT‒statistical features revealed that the recognition and perturbation information extraction performance of the proposed algorithm were significantly higher.
3. By comparing the performances of algorithms using other classification models with that using the GBDT classification model, it was concluded that the perturbation identification and perturbation information extraction performance of the proposed algorithm are significantly improved when GBDT is used for discrete time-series data classification.
4. In this study, only two production activities were considered: air-door and mine-car operation. These operations can cause abnormal fluctuations in mine wind-velocity data. In actual production, production activities such as cage lifting can also interfere with mine wind-velocity data. In the next study, the proposed algorithm will be improved through additional experiment, to enable extraction of more production activity information and identification of more types of mine production activities based on abnormal fluctuations in wind-velocity data.
5. Compared with the latest research [2], the algorithm proposed in this paper has made progress in distinguishing the abnormal fluctuation of wind velocity data caused by different disturbance sources in the mine
6. The algorithm framework proposed in this paper is not only suitable for the identification of mine wind speed disturbance activities, but also suitable for the identification of the causes of abnormal fluctuations in monitoring data in other fields.

## Supporting information

S1 Data (ZIP)
